# Loss of Uhrf1 in neural stem cells leads to activation of retroviral elements and delayed neurodegeneration

**DOI:** 10.1101/gad.284992.116

**Published:** 2016-10-01

**Authors:** Vidya Ramesh, Efil Bayam, Filippo M. Cernilogar, Ian M. Bonapace, Markus Schulze, Markus J. Riemenschneider, Gunnar Schotta, Magdalena Götz

**Affiliations:** 1 Institute for Stem Cell Research, Helmholtz Center Munich, 85764 Neuherberg, Germany;; 2 Physiological Genomics, Helmholtz Center Munich, 85764 Neuherberg, Germany;; 3 Department of Molecular Biology, Biomedical Center, Ludwig-Maximilians-University, 82152 Munich, Germany;; 4 Department of Functional and Structural Biology, University of Insubria, 21052 Busto Arsizio, Italy;; 5 Department of Neuropathology, Regensburg University Hospital, 93053 Regensburg, Germany;; 6 Munich Center for Integrated Protein Science (CiPS^M^), 81377 Munich, Germany;; 7 SyNergy, Excellence Cluster Systems Neurology, University of Munich, 81377 Munich, Germany

**Keywords:** neural stem cells, neuronal differentiation, IAP, Tet, 5hmC

## Abstract

In order to understand whether early epigenetic mechanisms instruct the long-term behavior of neural stem cells (NSCs) and their progeny, we examined *Uhrf1* (ubiquitin-like PHD ring finger-1; also known as Np95), as it is highly expressed in NSCs of the developing brain and rapidly down-regulated upon differentiation. Conditional deletion of *Uhrf1* in the developing cerebral cortex resulted in rather normal proliferation and neurogenesis but severe postnatal neurodegeneration. During development, deletion of *Uhrf1* lead to global DNA hypomethylation with a strong activation of the intracisternal A particle (IAP) family of endogenous retroviral elements, accompanied by an increase in 5-hydroxymethylcytosine. Down-regulation of Tet enzymes rescued the IAP activation in *Uhrf1* conditional knockout (cKO) cells, suggesting an antagonistic interplay between *Uhrf1* and *Tet* on IAP regulation. As IAP up-regulation persists into postnatal stages in the *Uhrf1* cKO mice, our data show the lack of means to repress IAPs in differentiating neurons that normally never express *Uhrf1*. The high load of viral proteins and other transcriptional deregulation ultimately led to postnatal neurodegeneration. Taken together, these data show that early developmental NSC factors can have long-term effects in neuronal differentiation and survival. Moreover, they highlight how specific the consequences of widespread changes in DNA methylation are for certain classes of retroviral elements.

## Abstract (precis)

Ramesh et al. show that conditional deletion of Uhrf1 in the developing cerebral cortex resulted in global DNA hypomethylation with a strong activation of the intracisternal A particle (IAP) family of endogenous retroviral elements. The high load of viral proteins and other transcriptional deregulation ultimately lead to postnatal neurodegeneration.

Neurogenesis is a complex process comprising several critical steps from proliferation of stem and progenitor cells to their differentiation and maturation into neurons. Each step of the neurogenic cascade is under tight transcriptional and post-transcriptional control and needs to be coordinated with the next differentiation step. Several transcription factors have been identified with key roles in specifying neurogenic fate in neural stem or progenitor cells, such as proneural factors Ascl1 and Ngn2, homeobox transcription factors Pax6 and Dlx, and many others (Schuurmans and Guillemot 2002; Wilkinson et al. 2013). These factors have early effects on neural stem cell (NSC) behavior and neurogenesis via transcriptional regulation. However, much less is known regarding whether and how mechanisms acting early in NSCs may exert effects only at later stages of neuronal differentiation or on postnatal gliogenesis. One of the key questions lies in unraveling the extent to which factors present at early developmental stages leave long-lasting traces on transcriptional regulation, affecting late stages of neuronal differentiation.

One mechanism by which gene regulation is modulated is via epigenetic modifications, such as DNA methylation (Tyssowski et al. 2014). DNA methylation is carried out by the Dnmt group of methyltransferases: Dnmt1, Dnmt3a, and Dnmt3b. Dnmt3 enzymes are de novo methyltransferases, whereas Dnmt1 is largely involved in maintaining DNA methylation in somatic cycling cells. DNA methylation modulates the expression of both genes and noncoding regions, such as transposable elements. In particular, it is involved in repressing transposable elements such as retrotransposons (REs). REs have been suggested to contribute to the evolution of complex brains and generation of neuronal diversity (Muotri et al. 2005; Singer et al. 2010). Thus, regulation of REs in neural development may be of particular importance, but its understanding is yet in its infancy.

Recent studies have shown that Uhrf1 (ubiquitin-like PHD ring finger-1; also known as Np95) acts as an important adapter for Dnmts (Meilinger et al. 2009; Berkyurek et al. 2014). Uhrf1 has been shown primarily to interact with Dnmt1, thus playing a major role in the maintenance of DNA methylation in proliferating cells. Uhrf1 also possesses a RING finger domain, which has been linked to histone and protein ubiquitination and turnover (Citterio et al. 2004; Liu et al. 2013; Nishiyama et al. 2013; Qin et al. 2015). Moreover, Uhrf1 has also been involved in establishing and reorganizing heterochromatin sites (Papait et al. 2008; De Vos et al. 2014). In embryonic or skin stem cells, *Uhrf1* is expressed during proliferation and down-regulated upon differentiation (Sharif et al. 2007; Sen et al. 2010). It has also been described to play a key role in regulatory T-cell differentiation and cancer formation and progression (Hervouet et al. 2010; Tien et al. 2011; Babbio et al. 2012; Obata et al. 2014; Pacaud et al. 2014).

Interestingly, in our genome-wide expression analysis of NSCs in the developing and adult forebrain (Pinto et al. 2008; Beckervordersandforth et al. 2010), we identified *Uhrf1* to be highly enriched in embryonic and adult NSCs. This was observed in contrast to *Dnmt1*, which is expressed in both NSCs and their differentiated progeny (Goto et al. 1994; Inano et al. 2000). Thus, we considered it an excellent candidate to investigate whether an early NSC factor can exert long-term effects on neurogenesis.

We found that early deletion of *Uhrf1* in cerebral cortex NSCs leads to neurodegeneration largely at postnatal stages in this region. Furthermore, we observed major changes in DNA methylation marks with little impact on gene expression and no change in cell fate. The most striking changes that we observed were on specific endogeneous retroviral elements (ERVs); namely, intracisternal A particle (IAP). We could determine the underlying mechanism of IAP regulation in this system, uncovering an antagonistic interplay between Uhrf1 and the Tet machinery on the regulation of specific ERV elements.

## Results

### Uhrf1 is expressed in embryonic and adult neural stem and progenitor cells

**Figure 1.**
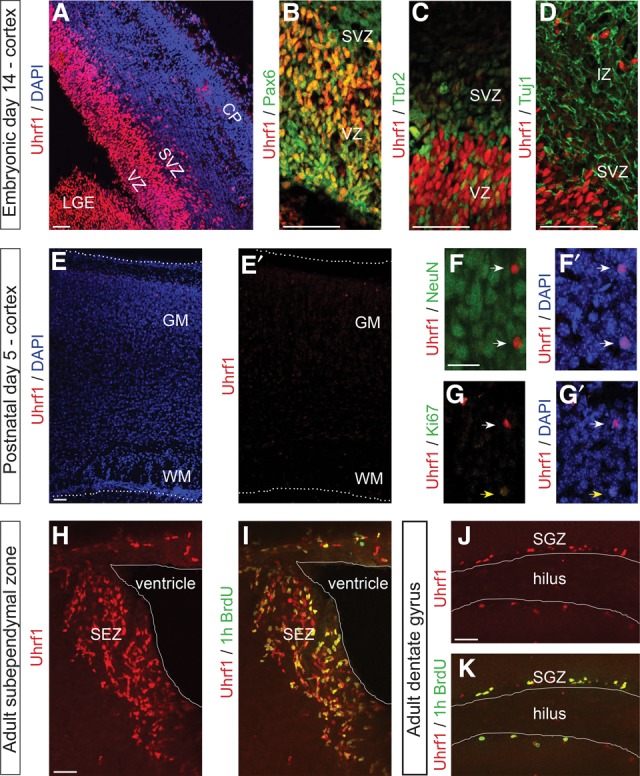
Immunostaining for Uhrf1 in the telencephalon from embryonic to adult stages. Confocal images of coronal sections of an E14 telencephalon (*A*–*D*) and a P5 cerebral cortex (*E*–*G*) as well as the subependymal zone (SEZ) (*H*,*I*) and dentate gyrus (*J*,*K*) of 2-mo-old mice stained as indicated. In *F* and *G*, white arrows indicate nuclei positive for only Uhrf1, while yellow arrows indicate Uhrf1/Ki67-double-positive nuclei. BrdU in *I* and *K* was given 1 h prior to analysis. Bars: *F*,*G*, 25 µm; all other panels, 50 µm. (IZ) Intermediate zone; (GM) gray matter; (WM) white matter; (LGE) lateral ganglionic eminence; (SGZ) subgranular zone.

We first performed a comprehensive analysis of *Uhrf1* expression at the peak of neurogenesis (embryonic day 14 [E14]) in the developing cerebral cortex. Uhrf1 immunoreactivity was very strong in the ventricular zone (VZ), where NSCs and progenitors are located (Fig. 1A). However, it was virtually absent in the intermediate zone and cortical plate (CP) of the dorsal telencephalon (Fig. 1A). Uhrf1 immunoreactivity clearly colocalized with the NSC marker Pax6 in the developing cerebral cortex (Fig. 1B). Thus, Uhrf1 is present early in the neurogenic lineage; namely, in Pax6^+^ neural stem and progenitor cells. It is rapidly down-regulated in the next stage of the lineage in Tbr2^+^ transit-amplifying progenitors in the subventricular zone (SVZ) (Fig. 1C). Uhrf1 is also not detectable in differentiating Tuj1^+^ neurons located in the CP (Fig. 1D). At early postnatal stages (e.g., postnatal day 5 [P5]), when neurogenesis has subsided in the cerebral cortex, Uhrf1 immunoreactivity was restricted to a few scattered cells (Fig. 1E,E′). These cells are likely glial progenitor cells, as some were proliferating and double-labeled for Ki67 (Fig. 1E,G). Similar to embryonic stages, Uhrf1 was not detectable in NeuN^+^ neurons (Fig. 1E,F).

In contrast to the few Uhrf1^+^ cells in the postnatal and adult cerebral cortex, Uhrf1 immunostaining was very prominent in both adult neurogenic niches—the subependymal zone (SEZ) and subgranular zone (SGZ) of the hippocampus (Fig. 1H–K). NSCs were detected by their label-retaining properties with the DNA analog BrdU. BrdU was given for 2 wk followed by a 2-wk chase without BrdU. Double stainings revealed that, in the SEZ, Uhrf1 was contained in a subset of NSCs, labeled by both BrdU and Gfap (Supplemental Fig. 1A–D). It was present in virtually all fast-proliferating transient-amplifying progenitors labeled by a 1-h pulse of BrdU (Fig. 1I,K) and many doublecortin^+^ neuroblasts (Supplemental Fig. 1E,F). We also performed quantitative RT–PCR (qRT–PCR) for *Uhrf1* in E14 NSCs (fluorescence-activated cell sorting [FACS] isolated with antibody against Prominin 1) and neurons (FACS isolated with antibody against PSA-NCAM) as well as adult tissue isolated from the adult SVZ and cerebral cortex white and gray matter (Supplemental Fig. 1G). Indeed, *Uhrf1* mRNA was already much lower in embryonic neurons and barely detectable in the adult cerebral cortex gray matter, where all the neurons reside. Conversely, glial cells in the white matter express *Uhrf1* to some degree, although at lower levels than the SVZ. Taken together, Uhrf1 is most prominently expressed during the early stages of the neurogenic lineage in both embryonic and adult brains.

### Uhrf1 deletion does not affect proliferation and cell fate in the developing cerebral cortex

**Figure 2.**
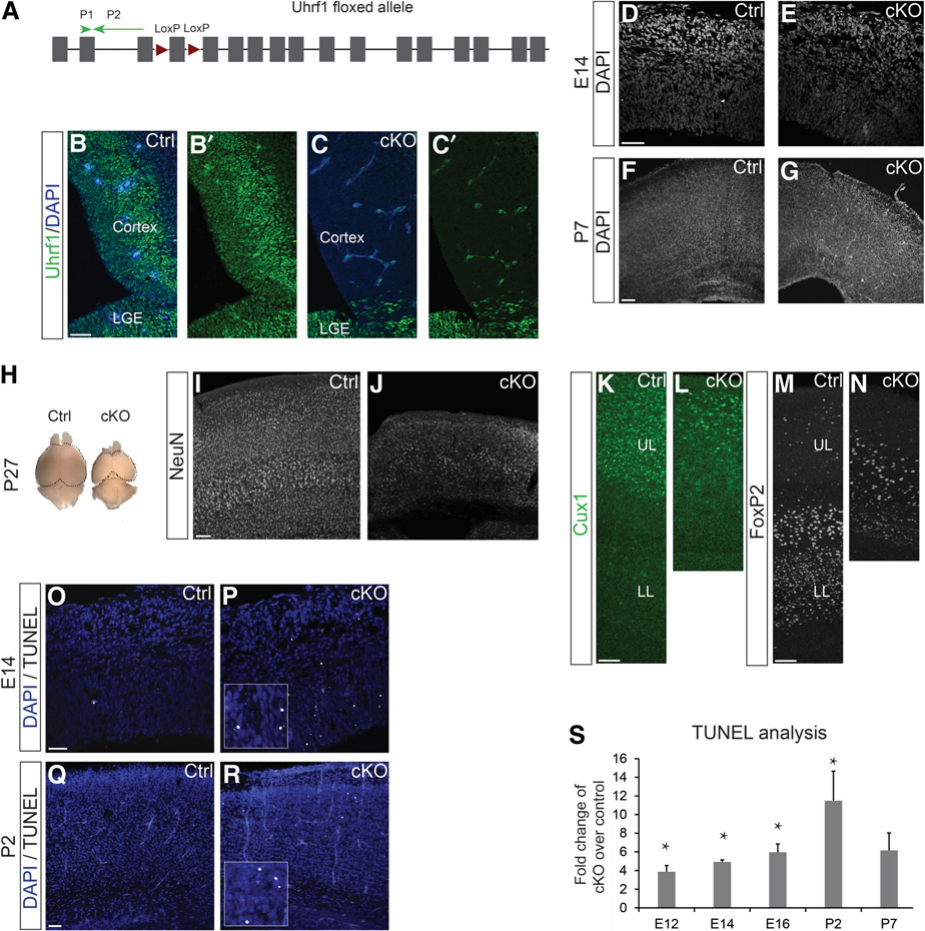
Cortex-specific *Uhrf1* cKO results in a delayed neurodegenerative phenotype. (*A*) Schematic drawing of a targeted allele in *Uhrf1* floxed mice obtained from EUCOMM (European Conditional Mouse Mutagenesis Program). Green arrows indicate the positions of qPCR primers, red arrows are loxP sites flanking exon 4 of *Uhrf1*, and gray bars are exons. (*B*,*C*) Confocal images of coronal sections of E12 control (Ctrl; *Emx1*^Cre+/−^ *Uhrf1*^fl/+^) (*B*) and E12 *Uhrf1* cKO (cKO; *Emx1*^Cre+/−^ *Uhrf1*^fl/fl^) (*C*) embryos showing a decrease of Uhrf1 immunostaining in the cKO cortex but not in the LGE. (*D*–*G*) Confocal images of coronal sections of E14 (*D*,*E*) and P7 (*F*,*G*) cortices in control and cKO stained with DAPI showing the comparable thickness and cellular architecture of the E14 cerebral cortex (*D*,*E*), while the P7 cKO cerebral cortex is reduced in thickness (*F*,*G*). (*H*) Macroscopic image of the full brain of a 1-mo-old control and cKO. Note the prominent reduction of the cerebral cortex hemispheres in the cKO at the *right*. (*I*–*N*) Confocal images of coronal sections of the cerebral cortex at 1 mo of age immunostained as indicated. (*I*,*J*) Images show pronounced thinning of the cKO cerebral cortex due to reduced NeuN^+^ neurons of all layers. (*K–N*) Images show reduction of both upper- and lower-layer neurons (Cux1 for the upper layers and FoxP2 for the lower layers). (*O*–*R*) Confocal images of coronal sections of the cerebral cortex at E14 (*O*,*P*) and P2 (*Q*,*R*) stained for TUNEL and nuclei stained with DAPI. The *insets* show TUNEL^+^ cells in the cKO. (*S*) Histogram showing fold change of TUNEL^+^ cells in cKO cerebral cortices over controls at the times indicated from E12 to P7. Error bars indicate standard error of mean. *n* = 4 or 5 for all stages except P7, where *n* = 2. (*) Significance with *P*-value of <0.05. Bar, 50 µm. (UL) Upper layers; (LL) lower layer.

To investigate the function of *Uhrf1* in neural stem and progenitor cells, we used the *Emx1*^Cre^ mouse line (Iwasato et al. 2000) to delete the floxed exon4 of *Uhrf1* (Fig. 2A) specifically in the dorsal telencephalon (Cappello et al. 2006). As reported previously, Cre-mediated deletion occurs around E10. Uhrf1 immunoreactivity was already almost completely lost at E12 in the cerebral cortex of *Emx1*^Cre/+^*Uhrf1*^−/−^ (referred to here as conditional knockout [cKO]) embryos, as compared with *Emx1*^Cre/+^ *Uhrf1*^+/−^ (referred to here as control) embryos (Fig. 2B,C). As expected by the region-specific recombination occurring only in the cerebral cortex, Uhrf1 immunostaining was not affected in the lateral ganglionic eminence (LGE) in cKO embryos (Fig. 2C).

To our surprise, the early deletion of *Uhrf1* had little effect on embryonic development, as the cerebral cortex had a normal size even at later stages, such as E14 (Fig. 2D,E) and P2 (Fig. 2Q,R). Moreover, numbers of NSCs and progenitors were comparable, as detected by Pax6 and Tbr2 immunostaining at E14 (Supplemental Fig. 2A–C,G,H). Quantifications of Pax6^+^ cells confirmed unchanged NSC numbers (Supplemental Fig. 2C). Likewise, the numbers of mitotic cells labeled by pH3 were not altered in *Uhrf1* cKO compared with controls (Supplemental Fig. 2D–F). Also, cortical neurons (labeled with Tbr1) were comparable in the CP of both genotypes, which also showed similar thicknesses (Supplemental Fig. 2I–L). Thus, in contrast to the previously reported role of *Uhrf1* in the proliferation of embryonic stem cells and regulatory T cells (Jeanblanc et al. 2005; Obata et al. 2014), it is largely dispensable for proliferation and major fate decisions in the developing dorsal telencephalon.

### Uhrf1 deletion results in neurodegeneration due to increased apoptosis at early postnatal stages

The first gross morphological phenotype after *Uhrf1* deletion became detectable at early postnatal stages (P7), when the thickness of the cKO cerebral cortex was reduced (Fig. 2F,G). At P27, the cerebral cortex size was significantly smaller not only macroscopically and in rostro-caudal extension (Fig. 2H) but also in thickness, as evident in coronal sections showing a severe reduction of NeuN^+^ neuronal layers (Fig. 2I,J). To understand whether *Uhrf1* plays a role in fate decisions, we investigated whether the neuronal composition of the remaining neurons was altered in the cKO cerebral cortex. However, both upper-layer neurons labeled by Cux1 immunostaining and lower-layer neurons labeled by FoxP2 were strongly reduced in number (Fig. 2K–N) in the cKOs, suggesting that neurons of all layers are similarly affected. In addition, the normal layer sequence was preserved, with upper-layer neurons on top of lower-layer neurons (Fig. 2K–N). In addition, we examined cortical layers at an earlier time point (E18) before the neurodegenerative phenotype was observed and did not observe any differences in cortical layering between controls and cKO cortices (Supplemental Fig. 2O,P), further suggesting that initial neuronal differentiation was unaffected.

We next examined cell death as a possible cause for this severe phenotype. Interestingly, while cortical thickness was not yet affected at E14, we already observed an increase in apoptotic TUNEL^+^ cells at this stage (Fig. 2O,P), which started at E12 (Fig. 2S). Moreover, both Pax6-positive and Pax6-negative cells were TUNEL-positive (Supplemental Fig. 2M,N), suggesting that multiple cell types are affected by the cell death. This fourfold to fivefold increase was maintained throughout embryogenesis but later peaked at early postnatal stages in cKO cerebral cortices, reaching 11-fold higher levels of cell death at P2 (Fig. 2Q–S). After this peak, the decrease in cortex thickness started to become visible from P5 on and was reproducibly noted at P7 (Fig. 2G; data not shown). Thus, loss of Uhrf1 at the onset of neurogenesis leads to an immediate increase in cell death that further escalates at the time of neuronal maturation during postnatal stages (Fig. 2S).

Immunostaining for glial cells with Olig2 and S100β showed a relatively comparable density at P5 and P27 in controls and *Uhrf1* cKO (Supplemental Fig. 3A,B,K,L). Reactive gliosis was detectable only after the peak of cell death, as indicated by an up-regulation of Gfap immunostaining at P27 (Supplemental Fig. 3I,J) that was absent at P5 (Supplemental Fig. 3C,D). Furthermore, we did not observe a premature Gfap expression or microglial activation in the embryonic cortex, suggesting that the embryonic cell death was insufficient to trigger reactive gliosis (Supplemental Fig. 3E–H).

Taken together, these data demonstrate that although Uhrf1 is expressed only in NSCs and is absent in neurons, it exerts long-term effects, as observed by the postnatal cellular degeneration.

### Activation of cellular stress genes with no transcriptional changes in cell fate genes

**Figure 3.**
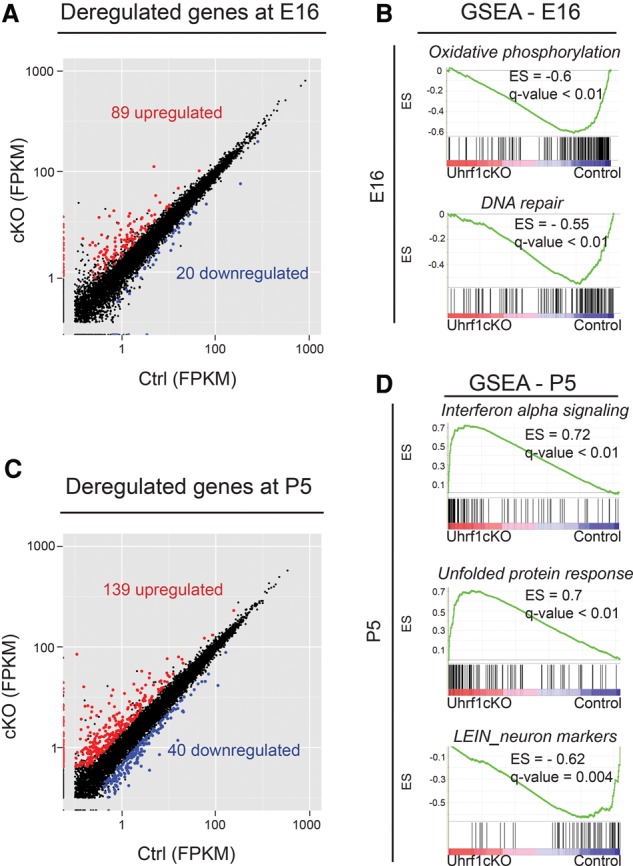
Transcriptional deregulation in *Uhrf1* cKO cerebral cortices at embryonic and postnatal stages. (*A*) Graph showing deregulated genes at E16 in the cerebral cortex of cKO. Red dots indicate genes with significantly increased expression, while blue dots indicate genes with significantly reduced expression in the cKO cerebral cortex compared with controls. Significantly regulated genes have a *P*-value of <0.05 and fold change of >2. The *X*-axis and *Y*-axis values are FPKM from 100-base-pair (bp) paired-end RNA-seq. (*B*) Graphs showing gene set enrichment analysis (GSEA) at E16 on hallmark and curated data sets. *Uhrf1* cKOs show underrepresentation of genes involved in oxidative phosphorylation and DNA repair. False discovery rate (FDR), <25%. (*C*) Graph showing RNA-seq results for coding genes from P5 cortices of cKOs compared with controls, as described for *A*. (*D*) Graphs showing GSEA at P5. (*Top*) Similar to E16, expression of genes involved in interferon α signaling is overrepresented also at P5 in cKO cortices. Genes involved in the unfolded protein response are also overrepresented (*middle*), and the LEIN neuron markers (curated data set) are underrepresented in expression in the P5 cKO (*bottom*). FDR, <25%.

In order to understand the molecular changes occurring during neurodegeneration in the *Uhrf1* cKO, we performed transcriptional profiling at E16 and P5. At E16, we detected surprisingly few significant changes of coding gene expression (*P*-value of <0.05; fold change of >2) between cKO and control cerebral cortices, with 89 up-regulated and 20 down-regulated genes (Fig. 3A). Interestingly, we observed 30% of deregulated genes to be derepressed in *Uhrf1* cKO (orange in Supplemental Table 1). Derepressed genes were defined as those having no expression in the control (<0.1 FPKM [fragments per million bases]) and activated in *Uhrf1* cKO (greater than twofold change). Notably, the deregulated genes did not contain any glial or other fate determinants, such as *Emx1/2*, *Tbr1/2*, *Ngn2*, or *Pax6* (Supplemental Table 1), consistent with the lack of fate changes observed by our histological analysis. However, we noted derepression of lineage-inappropriate genes such as those related to the germ cell lineage (green in Supplemental Table 1). Furthermore, in order to detect the transcriptional changes in cells expressing Uhrf1, we performed RNA sequencing (RNA-seq) specifically in the germinal zones of *Uhrf1* control and cKO cortices. We observed a mild transcriptional deregulation similar to that observed in the full cortex profiling (Supplemental Fig. 4A).

In order to detect whether any pathways were significantly changed upon lack of Uhrf1, we performed gene set enrichment analysis (GSEA). GSEA is a method that determines whether defined sets of genes are statistically significant between two biological states (Mootha et al. 2003; Subramanian et al. 2005). This analysis revealed underrepresentation of genes related to oxidative phosphorylation and DNA repair in the cKO (Fig. 3B). Interestingly, when we performed GSEA on the E16 VZ data set, we observed interferon signaling to be additionally overrepresented in the cKO as well as a similar trend for oxidative phosphorylation (Supplemental Fig. 4B).

These findings prompted us to analyze gene expression changes at a postnatal stage (P5) prior to the observed cortical thinning. Changes in gene expression between cKO and control cerebral cortices were slightly increased compared with embryonic stages, with 139 genes significantly up-regulated in expression, of which 96 genes were increased, 43 were derepressed, and 40 were down-regulated (*P*-value of <0.05; fold change of >2) (Fig. 3C; Supplemental Table 2). Interestingly, at this stage (P5), GSEA analysis revealed an underrepresentation of genes related to LEIN neuron markers (Fig. 3D), which are genes enriched in the adult mouse brain. These genes comprise many ion channels and other genes encoding for functional aspects of neurons. These data could suggest that cortical neurons in *Uhrf1* cKO are hampered in up-regulating genes involved in terminal neuronal differentiation pathways to physiological levels. Alternatively, the slight reduction in neurons in the P5 *Uhrf1* cKO cortex may be reflected by the decreased expression of these neuronal markers. In addition, many genes related to the unfolded protein response and interferon signaling were up-regulated in the cKO (Fig. 3D), indicative of cellular stress. Deregulation of such pathways has been described as leading to cell death (Sano and Reed 2013). Thus, our genome-wide expression analysis revealed aggravating changes in gene expression at postnatal stages, with several factors that could contribute to the neuronal death in the cKOs.

### Specific ERVs are activated in Uhrf1 cKOs

**Figure 4.**
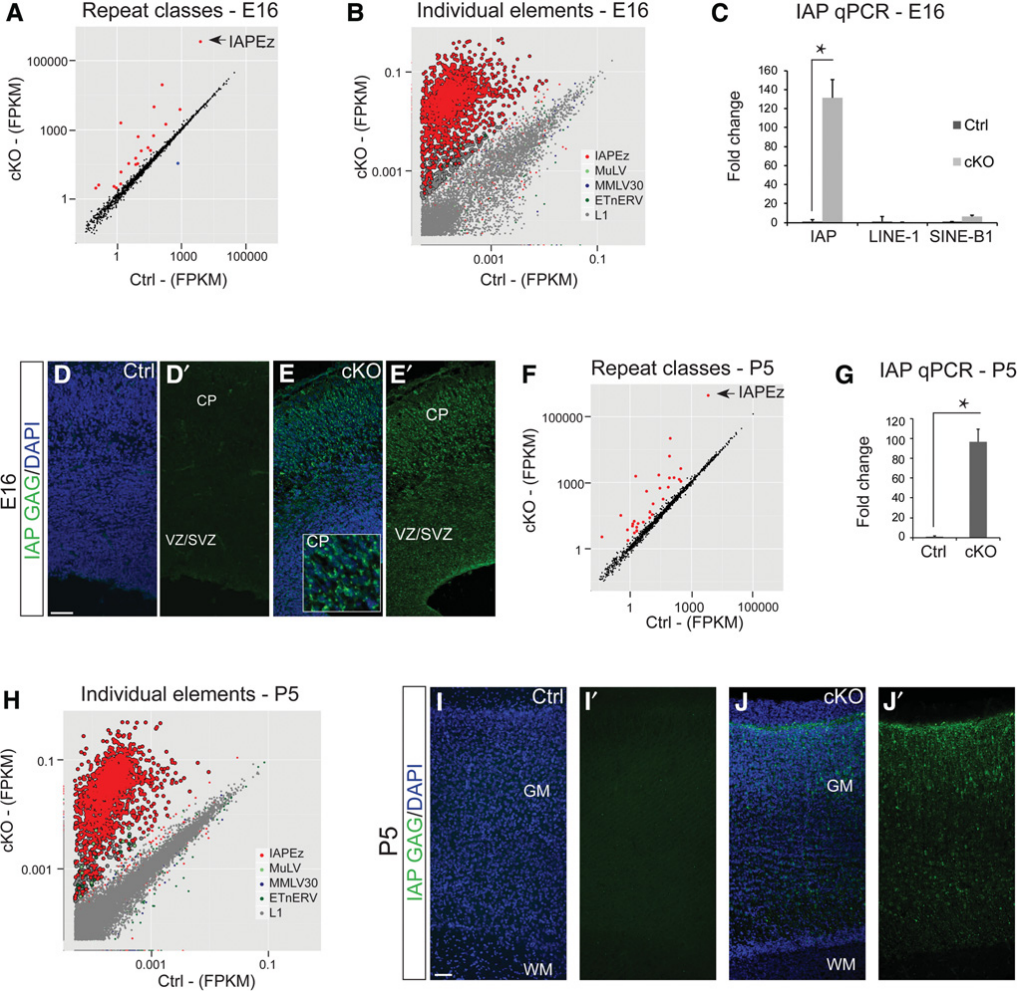
Activation of IAP retroviral elements in *Uhrf1* cKO cerebral cortices. (*A*) Graph depicting activation of classes of repeat elements in E16 cKO cerebral cortices. Red dots indicate repeat classes with significantly increased expression, and blue dots indicate repeat classes with significantly down-regulated expression in the cKO cerebral cortices compared with controls. The *X*-axis and *Y*-axis values are FPKM from 100-bp paired-end RNA-seq. (*B*) Graph depicting individual repeat elements in E16 cKO cerebral cortices. The *X*-axis and *Y*-axis values are FPKM from 100-bp paired-end RNA-seq. (*C*) RT-qPCR graph for IAP in E16 control and cKO cortices and L1 and SINE-B1 elements in E14 control and cKO cortices. (*) *P*-value = 0.01. *n* = 4 per condition. Error bars indicate standard error of mean. (*D*,*E*) Confocal images of coronal sections of E16 cortices of controls (*D*) and cKOs (*E*) immunostained for the Gag protein show a strong increase in immunoreactivitity in the cKO cortex (*E*). (*F*,*H*) Graph depicting activation of repeat classes (*F*) and individual repeat elements (*H*) in the P5 cKO cortex compared with the control cortex as described in *A* and *B*. (*G*) qPCR graph for IAP in P5 control and cKO cortices and L1 and SINE-B1 elements in P5 control and cKO cortices. (*) Significance with *P*-value of <0.0001. *n* = 4 for each condition. Error bars indicate standard error of mean. (*I*,*J*) Confocal images of coronal sections of P5 control (*I*) and cKO (*J*) cortices immunostained for the Gag protein, with high levels in the cKO cortex. Bar, 50 µm. (GM) Gray matter; (WM) white matter.

The up-regulation of interferon signaling upon *Uhrf1* deletion at both E16 and P5 raised a possible link to activation of REs (Chiappinelli et al. 2015). Thus, we explored the RNA-seq data set mentioned above for expression of REs. We observed the strongest up-regulation of the IAPEz family in the E16 cortex of cKO, compared with control (Fig. 4A). Other classes of IAPs and other ERVs were also up-regulated to a lesser degree (Supplemental Fig. 5). We observed a bias for longer IAP copies (Supplemental Fig. 6A). Up-regulation of IAP transcripts is not mediated through transcriptional up-regulation of genes, as many IAP copies are intergenic (Supplemental Fig. 6B,C). We also detected only subtle effects on the transcriptional activity of neighboring genes (Supplemental Table 3). From other classes of REs, only some individual LINE-1 (L1) and MuLV elements were up-regulated in the cKOs (Fig. 4B). The IAPEz family is the predominant subclass of IAP elements in the mouse genome and has been described as active and able to integrate in new places in the genome (Dewannieux et al. 2004). In order to investigate whether all IAPEz copies in the genome were up-regulated, we analyzed the expression of individual loci and found essentially all IAPEz elements to be up-regulated (Fig. 4B, red dots). RT-qPCR for the 5′ untranslated region (UTR) of IAP, which captured the majority of the IAP elements present in the mouse genome, revealed ∼130-fold increase in expression in the cKO cortices (Fig. 4C). This analysis further confirmed the specificity of IAP but not L1 and SINE-B1 repeat element activation (Fig. 4C). In order to ascertain whether the up-regulated IAP mRNA is being translated and identify which cell types are expressing IAP, we performed immunostaining for the Gag region of IAP. IAP mRNA levels were already elevated in cKOs at E12 and E14 (Supplemental Fig. 7A,D), while Gag protein levels became detectable only at E14 (Supplemental Fig. 7B,C,E,F). Gag was strongly up-regulated in the *Uhrf1* cKO cerebral cortex but not in the LGE and control cerebral cortex, where Uhrf1 was not deleted (Fig. 4D,E; Supplemental Fig. 7F).

Strikingly, at postnatal stages 10 d later, IAP elements were still immensely up-regulated on mRNA (Fig. 4F–H) and protein (Fig. 4I,J) levels in the cKO cortex. These data demonstrate that the IAP activation in cKO is not compensated for in cells that do not express Uhrf1 (neurons or glia), thus highlighting the importance of Uhrf1 in permanent silencing of IAPs.

### Uhrf1 cKO exhibit global hypomethylation

**Figure 5.**
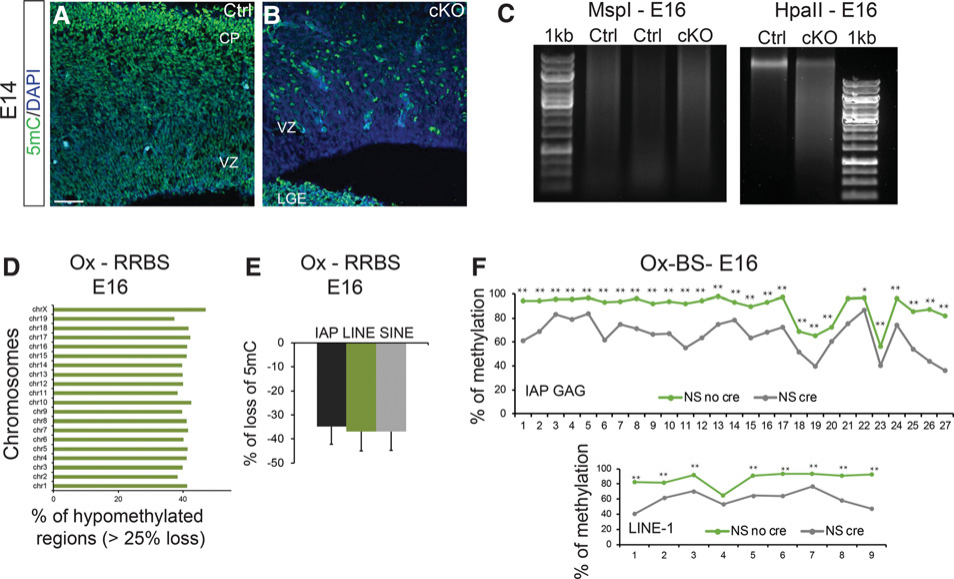
Changes in DNA methylation in *Uhrf1* cKO cerebral cortices. (*A*,*B*) Confocal images of coronal sections of E14 control (*A*) and cKO (*B*) cerebral cortices stained for 5mC and DAPI. (*B*) Note that 5mC is much reduced in the cKO cortex but not LGE. (*C*) Restriction digests of E16 genomic DNA from controls and *Uhrf1* cKO digested with HpaII and MspI and analyzed on a 0.5% agarose gel. (1 kb) One-kilobase DNA ladder. Note that both enzymes recognize the CCGG sequence; however, HpaII is unable to cut DNA when the internal cytosine is methylated. As much more DNA was cut in the *Uhrf1* cKO samples, this indicates less DNA methylation upon Uhrf1 deletion. (*D*,*E*) Ox-RRBS in E16 control and cKO cortices, with graphs depicting the percentage of regions hypomethylated (*X*-axis) per chromosome (*Y*-axis) (*D*) and the percentage of hypomethylation in each repeat element class (*E*). Error bars indicate standard error of mean between the values from each independent element from each class. (*F*) Ox-BS in E16 control and cKO cortices showing a loss of 5mC in *Uhrf1* cKO cortices. High-throughput sequencing was performed on the IAP Gag region and L1. The plot displays 27 CpGs present on IAP Gag loci, nine CpGs present on L1 loci, and their methylation status. The *Y*-axis indicates the percentage of methylation, and the *X*-axis indicates individual CpGs. (*) *P*-value ≤ 0.001; (**) *P*-value < 0.0001.

Having observed transcriptional deregulation and an activation of the IAP repeat elements, we sought to understand the underlying molecular mechanisms. As mentioned before, Uhrf1 is involved in maintaining global DNA methylation, and thus we examined levels of 5-methylcytosine (5mC) in cKO compared with controls. First, we used immunostainings for 5mC to detect any global alterations after *Uhrf1* deletion. Indeed, 5mC immunoreactivity appeared much reduced in the cKO cortex, but not in the LGE, compared with the controls (Fig. 5A,B). This phenotype was further corroborated by digestion with HpaII and MspI on genomic DNA from cKOs and controls (Fig. 5C). Both enzymes recognize and cut CCGG sites, but the HpaII enzyme does not cut when the internal cytosine is methylated. MspI showed no difference between controls and cKOs. However, the cKO cortex was more sensitive to HpaII as compared with controls (Fig. 5C), confirming loss of 5mC in cKOs. To identify specific hypomethylated genomic loci, we performed oxidative reduced representation bisulfite sequencing (Ox-RRBS) in E16 cKOs and controls. We detected a fairly uniform loss of 5mC, with all chromosomes exhibiting at least 25% loss of 5mC on nearly half of the loci, resulting in 850 hypomethylated promoters (Fig. 5D; Supplemental Fig. 8A). To investigate whether hypomethylated regions correlated with transcriptional activation, we overlapped data sets from Ox-RRBS and RNA-seq at E16. Surprisingly, reduced DNA methylation had little effect on transcription of genes, as only 0.2% of hypomethylated promoters in the E16 cKO cortex have significantly up-regulated transcripts (Supplemental Fig. 8A). Moreover, we also probed the data sets for 5mC levels in REs and detected a 35% loss on IAP, L1, and SINE-B1 elements (Fig. 5E). Additionally, we performed loci-specific oxidative bisulfite sequencing (Ox-BS) at the IAP *Gag* region and L1 and observed an overall 25% loss of 5mC, with some specific CpGs showing up to 40% loss of 5mC on both IAP and L1 (Fig. 5F). Examining L1 elements specifically, we found only few individual elements that showed up-regulated transcript levels despite a loss of 5mC (Supplemental Fig. 8B). Moreover, we specifically investigated 5mC levels at two L1 regulatory regions from RepBase (L1Md_F and L1VL1) and observed a similar loss of methylation (Supplemental Fig. 8C). These data suggest that the extent of L1 hypomethylation observed in *Uhrf1* cKO samples is insufficient to cause their transcriptional up-regulation (Supplemental Fig. 8C). Altogether, these data suggest that the loss of the 5mC mark on coding genes and L1 and SINE-B1 elements mostly does not translate into transcriptional changes. The only exception to this observation was on IAPs (Figs. 4A–C, 5E).

### Specific increase of 5-hydroxymethylcytosine (5hmC) on IAPs in Uhrf1 cKOs

**Figure 6.**
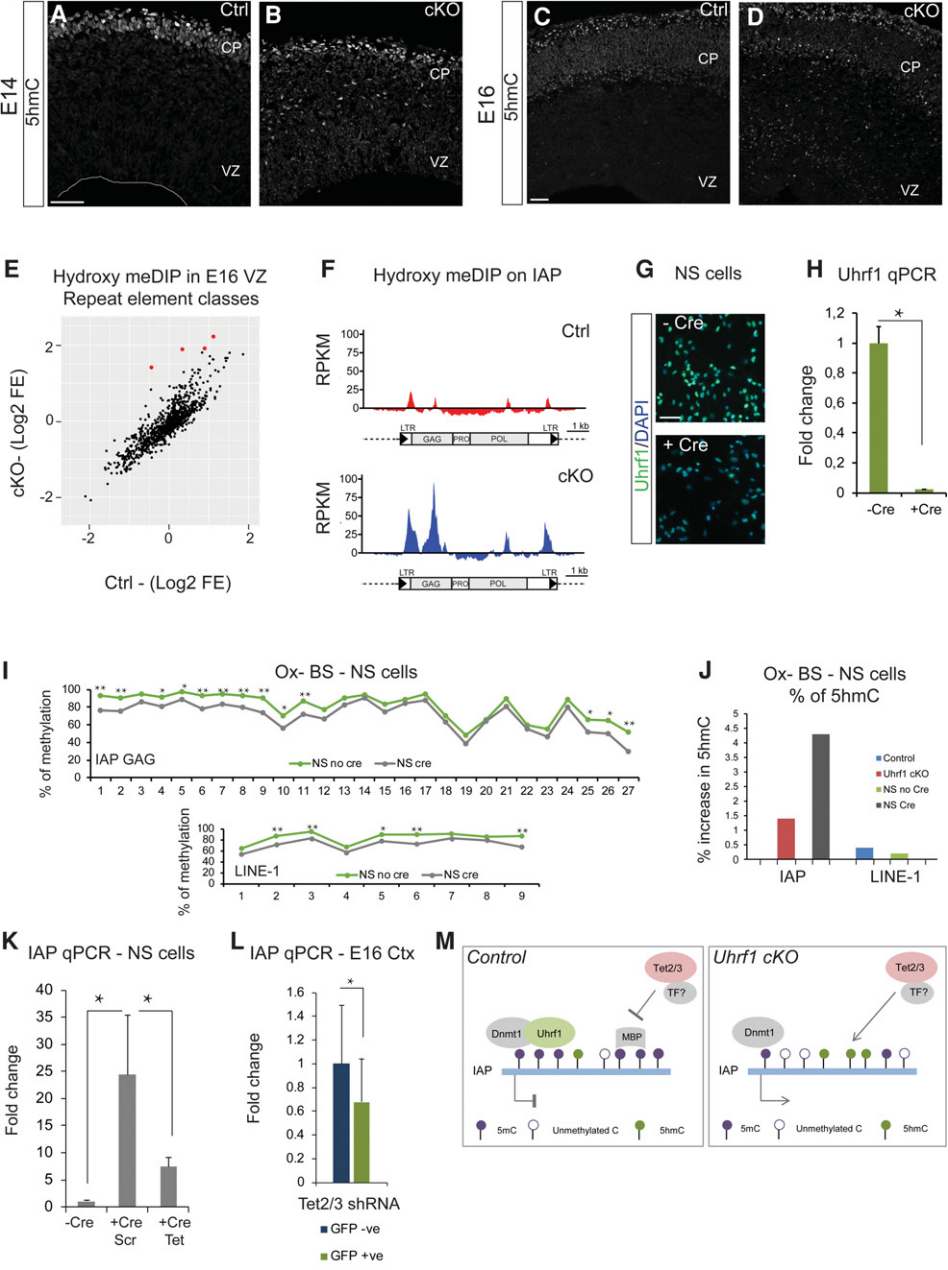
Mechanisms of IAP activation in the *Uhrf1* cKO cerebral cortices. (*A*–*D*) Confocal images of coronal sections of E14 (*A*,*B*) and E16 (*C*,*D*) control and cKO cortices immunostained for 5hmC. (*E*) Graph depicting repeat element classes in hmeDIP of E16 VZ tissue. The *X*-axis and *Y*-axis values are log_2_ fold enrichment for controls and cKOs from 50-bp single-end DNA sequencing. *P*-value < 0.05. Red dots indicate significantly up-regulated classes in the cKO with a greater than twofold change. (*F*) Plot of hmeDIP peaks on IAP in E16 VZ control and cKO. The *Y*-axis indicates RPKM (reads per kilobase per million mapped reads) values, and the *X*-axis indicates the IAP sequence. (*G*) Epifluorescence images of NSCs immunostained for Uhrf1 and DAPI in conditions without Cre and with Cre protein added to the cultures. (*H*) qPCR for *Uhrf1* showing strong down-regulation of *Uhrf1* mRNA 4 d after Cre protein addition to the cultures. (*) *P*-value = 0.02. *n* = 4 for −Cre; *n* = 8 for +Cre. Error bars indicate standard error of mean. (*I*) Graph indicating loss of 5mC on IAP Gag and L1 loci in *Uhrf1* floxed NSCs. High-throughput sequencing was performed on IAP and L1. The plot displays 27 CpGs present on IAP Gag loci, nine CpGs present on L1 loci, and their methylation status. (*) *P*-value ≤ 0.001; (**) *P*-value < 0.0001. (*J*) Graph indicating the percentage gain of 5hmC on IAP and L1 in *Uhrf1* floxed NSCs with and without Cre protein and E16 *Uhrf1* cKO cortical tissue compared with controls. The *Y*-axis indicates the percentage gain of 5hmC. (*K*) qPCR for IAP showing much reduced expression in the *Tet2/3* shRNA condition compared with scrambled shRNA. *Uhrf1* floxed NSCs were transfected with scrambled shRNA or *Tet2/3* shRNA and simultaneously treated with Cre protein. qPCR was performed after 4 d in vitro. (*) Significance. Unpaired *t*-test *P*-value of 0.02 for no Cre versus Cre/scrambled; paired *t*-test *P*-value of 0.04 for Cre/scrambled versus Cre/*Tet2,3* shRNA. *n* = 4 for each condition. Error bars indicate standard error of mean. (*L*) qPCR for IAP showing much reduced expression in the *Tet2/3* shRNA GFP-positive cells compared with GFP-negative cells in vivo. *Uhrf1* cKO embryos were electroporated at E13, and the GFP-positive and GFP-negative cells were isolated by FACS 3 d later. (*) *P*-value = 0.02, paired *t*-test. Error bars indicate standard error of mean. Paired statistical analysis was performed on these data, since the cells were from the same embryo either transfected or untransfected. Moreover, each pair of control and cKO showed a similar trend, with high variation between replicate pairs. (*M*) Model of IAP regulation in controls and *Uhrf1* cKOs. In controls, IAP is highly methylated by Uhrf1, preventing access for Tet and possibly certain transcription factors to the IAP loci. In *Uhrf1* cKOs, the absence of Uhrf1 leads to reduced 5mC, increased 5hmC and Tet activity, and possibly increased access for transcription factors. This allows transcription from the IAP locus. Bar, 50 µm. (TF) Transcription factor; (MBP) methylated DNA-binding protein.

DNA modifications such as 5hmC have been described as involved in transcriptional activation (Wu et al. 2011). Moreover, Uhrf1 has been shown to bind 5hmC (Bostick et al. 2007; Hashimoto et al. 2008; Frauer et al. 2011). Thus, we examined 5hmC by immunostaining. As had been described previously (Hahn et al. 2013), 5hmC immunoreactivity was restricted mostly to the neuronal layers in the developing cerebral cortices of control E14 and E16 embryos (Fig. 6A,C). Notably however, we observed very pronounced staining in the VZ/SVZ of the cKO cortices (Fig. 6B,D). In order to identify specific changes of 5hmC on genomic loci, we performed hydroxymethylated DNA immunoprecipitation (hmeDIP) followed by next-generation sequencing. We used the VZ fraction of E16 cKO and control cerebral cortices, enriching for the up-regulated 5hmC (Fig. 6D). Strikingly, the strongest increase of 5hmC in *Uhrf1* cKOs was present on the IAP class of repeat elements (log_2_ fold enrichment of >2; *P*-value of <0.05) (Fig. 6E, red dots). Furthermore, the subtypes of IAPs with increased 5hmC were identical to those with elevated transcript levels (Supplemental Figs. 5, 8D). Cumulative coverage plots revealed that the increase of 5hmC on IAPs was specific to the long terminal repeat (LTR) regions, which act as promoters in IAP, and the SHIN region within the *Gag* domain. The SHIN region is a sequence critical for IAP silencing in embryonic stem cells (Sadic et al. 2015). Interestingly, 5hmC was unchanged on other REs (Fig. 6E).

We hypothesized that the gain of 5hmC on IAP could be due to increased access for Tet enzymes when Uhrf1 is absent. Tet enzymes can actively convert 5mC to 5hmC (Tahiliani et al. 2009; Ito et al. 2010). This accumulation of 5hmC could in turn lead to the transcriptional activation of IAPs observed in the cKO. In order to test this hypothesis, we used previously tested shRNAs to knock down Tet enzymes (Hahn et al. 2013) in *Uhrf1* cKO NSCs. We generated expandable NSC cultures from E13 Uhrf1 floxed cortices (Conti et al. 2005; Onorati et al. 2011). The *Uhrf1* floxed NSCs were treated with recombinant Cre protein to induce Uhrf1 deletion, resulting in a 20-fold reduction of *Uhrf1* mRNA and protein after 4 d (Fig. 6G,H). Consistent with the above-described in vivo data, we observed loss of 5mC upon Cre transduction (Supplemental Fig. 9A,B) and IAP mRNA activation upon Cre induction but not that of L1 and SINE-B1 elements (Supplemental Fig. 9C). Moreover, using loci-specific Ox-BS, we observed an overall 10%–15% loss of 5mC on IAP, with some CpGs showing up to 15% loss of 5mC. This rather small loss of 5mC is possibly due to the partial Cre transduction of *Uhrf1* floxed NSCs. However, this loss of 5mC was accompanied by a 4.3% gain of 5hmC on IAP but no gain on L1 (Fig. 6J, green bar). We compared this increase in 5hmC in NSCs with that of the in vivo E16 cortical tissue and could observe only a 1.4% increase in 5hmC in *Uhrf1* cKOs, with no increase on L1 (Fig. 6J, blue bar). These data suggest that, although we observed a slight increase in 5hmC on IAP in the in vitro and in vivo *Uhrf1* cKO, the relative abundance of this 5hmC might be quite low (Fig. 6J). Thus, we observed many aspects of the in vivo phenotype to be recapitulated in the in vitro system upon loss of Uhrf1 in NSCs.

Among the Tet enzymes, *Tet2* and *Tet3* are the highest expressed in the embryonic cortex, playing important roles in neurogenesis, whereas *Tet1* has a very low expression (Hahn et al. 2013). We transfected shRNAs against *Tet2* and *Tet3* (Hahn et al. 2013) in the *Uhrf1* floxed NSCs, simultaneously inducing Cre-mediated deletion of *Uhrf1*. qPCR for IAP transcripts showed threefold to fourfold lower activation upon Tet down-regulation as compared with the scrambled shRNA (Fig. 6K), suggesting an important contribution of Tet enzymes to IAP expression. We performed paired analysis of this experiment, since the transfections and Cre transductions were performed on the same pool of cells. Each pair of control and cKO showed a similar trend, although there was a high variation between replicates, possibly due to the timing of IAP activation against the timing of *Tet2/3* knockdown. In order to corroborate these results in vivo, we performed in utero electroporation of Tet2/3 shRNA into E13 *Uhrf1* cKO embryos followed by FACS of the GFP-positive and GFP-negative cells at E16. IAP qRT–PCR and paired statistical analysis revealed ∼25% less IAP mRNA levels in the Tet electroporated GFP-positive cells in comparison with the surrounding nonelectroporated GFP-negative cells (Fig. 6L). These data suggest that Tet2 and Tet3 are mainly responsible for the observed IAP activation in vivo.

## Discussion

### Global 5mC and 5hmC changes do not affect proliferation and cell fate in the developing cerebral cortex

Loss of Uhrf1 in cortical NSCs led to a mild deregulation of coding genes with no change in major fate determinants of neurogenesis and gliogenesis. Consistent with this, we found no obvious difference in NSC proliferation and differentiation, contrary to the findings in other organs and cell types (Sharif et al. 2007; Obata et al. 2014). In particular, numbers of NSCs (shown by pH3 and Pax6), neurons (labeled by Tbr1), and glia (Gfap, Olig2, and S100β) were comparable. This is particularly intriguing, as the loss of Uhrf1 resulted in severe DNA hypomethylation, demonstrating that even severely hypomethylated NSCs are well capable of proliferation and differentiation. Moreover, we did not observe any premature onset of gliogenesis or glial differentiation by Gfap, S100β, or Olig2 immunostaining. Although we observed a mild up-regulation of Gfap in the E16 VZ RNA-seq, we could not detect any transcripts via RT-qPCR, suggesting a very low activation. This is contrary to the phenotype in the DNMT1 cKOs that show strong derepression of the *Gfap* promoter and a premature increase in Gfap and S100β cells in various regions of the embryonic CNS, including the cerebral cortex (Fan et al. 2005). As this is not the case in *Uhrf1* cKOs, these data imply other adaptor proteins for Dnmt1. Moreover, the function of *Uhrf1* in the developing cerebral cortex differs from its effects in other cells, revealing its highly specific role in transcriptional regulation in this context.

Indeed, this conclusion is further corroborated by comparison of gene expression changes in this *Uhrf1* cKO and the *Dnmt1* cKO (Hutnick et al. 2009). This was possible because both of these genes were deleted with an *Emx1*-directed Cre, and transcriptional profiling was performed at the same stage (P5) (Hutnick et al. 2009). This comparison revealed only 16%–20% overlap in the genes, with significant changes in expression (Supplemental Fig. 10). Moreover, only three of the common up-regulated genes are hypomethylated in the *Uhrf1* cKO at E16 (Supplemental Table 4, genes highlighted in red). This is also the case when examining earlier stages (E16) by RNA-seq and Ox-RRBS, as only four of 186 up-regulated genes (fold change of >2; *P*-value of <0.05) had reduced methylcytosine levels in the cKO cerebral cortex (Supplemental Fig. 8A). These results demonstrate clearly that the rather prominent loss of DNA methylation in the *Uhrf1* cKO cortex has only minor effects on gene expression and shows no detectable effects on major cell fate decisions.

### Uhrf1 is critical for IAP repression

The changes in DNA modification, however, lead to transcriptional activation on only one set of genomic loci: the IAP elements. IAP elements were activated as early as E12 and reached massive transcript and protein levels at later stages. Intriguingly, even after several weeks, the IAP activation could not be counteracted, demonstrating the need for Uhrf1 in repressing the transcription of these specific ERVs at early stages. It is important to note that *Uhrf1* tamoxifen-inducible deletion of the whole embryo did not lead to a major overall increase in IAPs (Sharif et al. 2016). Our study clearly demonstrates that, in the developing cerebral cortex, loss of Uhrf1 leads to strong IAP activation. Thus, Uhrf1 has clear cell type-specific roles in ERV regulation. It is interesting to note that although loss of 5mC occurred on several repeat classes, only IAP elements were transcriptionally activated. We hypothesize that this could be due to differences in chromatin landscape between different repeat classes. We speculate that reduced DNA methylation compromises the heterochromatic state of IAP elements in such a way that specific transcription factors and Tet2/3 can bind these elements and activate transcription. Moreover, the regulation of IAP transcription by Uhrf1 could be direct, as it has been demonstrated recently that Uhrf1 can bind IAP (Sharif et al. 2016).

In this regard, the *Uhrf1* cKO phenotype is very comparable with the *Dnmt1* cKO cerebral cortex that also shows persistent up-regulation of IAPs into postnatal stages (Hutnick et al. 2009). This is rather surprising, as other members of the Dnmt family of DNA methyltransferases, such as *Dnmt3a* and *Dnmt3b*, are expressed in the neurons of the cerebral cortex (Feng et al. 2005), unlike Uhrf1. These data suggest that, even in the absence of Dnmt1/Uhrf1 machinery, the other Dnmts are unable to repress the IAP elements. Indeed, conditional deletion of *Dnmt*s in neurons showed no degenerative phenotype (Fan et al. 2001; Feng et al. 2010), consistent with the interpretation that once repression of these elements is initiated in NSCs, it is retained in the progeny. Thus, differentiated cells such as adult neurons probably do not convert their 5mC into 5hmC on repeat elements to cause their activation.

We observed a specific increase in 5hmC on IAPs in the cKO. We confirmed our hypothesis that the increased 5hmC could arise from Tet activity by down-regulating *Tet* enzymes in *Uhrf1* cKO and partially rescuing IAP activation. Thus, our data suggest that, in NSCs deleted for *Uhrf1*, 5mC levels are reduced, and Tet2/3 access to IAPs is probably increased. This in turn leads to increased 5hmC on IAP LTR and *Gag* regions, culminating in IAP activation (Fig. 6M). It is likely that the accumulation of 5hmC on IAP promoter regions facilitates binding of certain transcription factors, allowing transcription from IAP loci.

### Postnatal neurodegeneration in Uhrf1 cKOs

The neurodegenerative phenotype that we observed in the *Uhrf1* cKO could be linked primarily to loss of DNA methylation, IAP activation, and the transcriptional deregulation of genes. First, up-regulation of IAP elements and global DNA demethylation have been associated with neurodegenerative and neuroinflammatory diseases (Perron and Lang 2010; Lu et al. 2013). Furthermore, overexpression of a human ERV, HERV-K, is sufficient to cause neurodegeneration (Li et al. 2015). Moreover, the increasing load of the Gag protein also seems to trigger an unfolded protein response in the *Uhrf1* cKO cortex (seen in RNA-seq at P5 but not E16), which often precedes neuronal death. Finally, we also saw clear mediators of cell death coming up at P5, such as p75, demonstrating the additional activation of death pathways not yet detectable at E16. Moreover, IAP activation has also been observed in the *Dnmt1* cKO of the cerebral cortex, which also results in a neuronal death phenotype (Hutnick et al. 2009). However, interestingly, in the *Setdb1* cKO in which IAP is only mildly up-regulated, neuronal death was not observed in the cortex (Tan et al. 2012).

In the *Uhrf1* cKO, cell death aggravates with time, exhibiting a progressive phenotype that is already detectable at early stages. We suggest two main mechanisms as causes of the cell death culminating in neuronal reduction at postnatal stages: (1) The phenotype deteriorates by additive deregulation of genes, and (2) some phenotypes are not critical at earlier stages but become deleterious later when neurons become functional. In regard to the first option, we noted the GSEA term “Lein neuron markers” to be decreased in the postnatal but not embryonic cKO. This term comprises terminal maturation genes, many of which encode ion channels and receptors, all highly relevant for the physiological function of neurons. These data suggest that up-regulation of neuronal maturation genes could be impaired in the *Uhrf1* cKO or that neurons die preferentially at this stage of differentiation.

In regard to the second cause, the significant impairment of genes involved in oxidative phosphorylation in the mitochondria, observed at embryonic stages, may have consequences mostly later. Mitochondrial dysfunction has been described as causing cellular stress and cell death in adult neurons and neurodegenerative disease (Johri and Beal 2012). Interestingly, deletion of the succinate dehydrogenase subunit D gene in cortex progenitors resulted in neuronal death only at postnatal stages (Diaz-Castro et al. 2015). This is consistent with the concept that both stem and progenitor cells and young neurons are more resistant to defects in mitochondria oxidative phosphorylation pathways as opposed to postnatal neurons. Moreover, RE-mediated activation of the unfolded protein response has been shown previously to induce apoptosis in B cells (Pasquarella et al. 2016). Last, up-regulation of interferon signaling can be triggered by retroviral activation and was linked to neuronal death (Chen et al. 2014), suggesting multiple pathways converging on the cell death phenotype. It is also important to note here that IAP activation could lead to transposition of IAP into new genomic loci, possibly contributing to the cell death phenotype. Thus, our cKO model provides a rather intriguing model for neurodegeneration, highlighting the time when neurons become vulnerable to mechanisms implicated in neurodegenerative disease, such as mitochondrial dysfunction and unfolded protein response.

### Conclusions

Our study has identified a rather uncharacteristic role for a NSC factor in controlling terminal neuronal differentiation and survival, which manifests at postnatal stages. This allowed unraveling of some key insights. First, Uhrf1 is a critical factor to maintain global DNA methylation in cortical NSCs and progenitors. Loss or gain of methylation marks on gene promoters is not deterministic for mRNA expression. However, Uhrf1 has a more specific role in the regulation of IAP retroviral elements via active maintenance of their DNA methylation and silencing. Second, we described a previously unknown finding in which loss of methylation on these IAPs leads specifically to the recruitment of active demethylation machinery (Tet enzymes), resulting in their activation (Fig. 6M).

A further intriguing concept is that a NSC-specific transcriptional regulator sets the stage for neuronal function or maintenance, occurring several weeks later. Our study shows that key factors, such as *Uhrf1*, expressed exclusively at early stages in the lineage, have long-reaching consequences into later stages of neuronal differentiation and survival.

## Materials and methods

All animals were kept in the animal facility of the Helmholtz Center Munich. The day of the vaginal plug was counted as E0. All experimental procedures were performed in accordance with German and European Union guidelines. The mouse lines used in the experiments are *Emx1*^Cre^ (Iwasato et al. 2000) and *Uhrf1*^fl^ (European Conditional Mouse Mutagenesis Program [EUCOMM]).

### Tissue processing

Brains from embryos were fixed by immersion for 2–4 h in 4% (w/v) paraformaldehyde in phosphate-buffered saline (PBS). Brains from postnatal mice were first perfused with the same fixative followed by immersion for 1 h. Following fixation, the brains were washed in PBS and immersed in 30% (w/v) sucrose in PBS. Embryonic tissue was then embedded in Tissue-Tek, frozen on dry ice, cut (cryostat, 14–16 μm thick), and stored at −20°C. Postnatal tissue was cut on a vibratome to 70 μm thick, and the sections were stored in PBS.

### Immunohistochemistry and image analysis

Frozen sections were thawed and washed in PBS. Both frozen and floating sections were incubated with blocking solution (0.01% Triton X, 10% normal goat serum in PBS) followed by primary antibodies, and the nuclei were visualized by DAPI. For methylation stainings, antigen retrieval was performed with 2 N HCl for 30 min followed by a 30-min incubation with 100 mM Tris-HCl (pH 8.0). Sections were analyzed using an Olympus Axioplan2 confocal laser-scanning microscope. Post-image processing in regard to brightness and contrast was carried out where appropriate to improve visualization in a pairwise manner. The following primary antibodies were used: mouse anti-Pax6 (1:200; Millipore), chick anti-Tbr2 (1:200; Millipore), mouse anti-Tuj1 (1:500; Sigma), mouse anti-NeuN (1:500; Millipore), rat anti-Ki67 (1:200; BD Bioscience), rabbit anti-IAP Gag (1:1000; a kind gift from Bryan Cullen, Duke University), chick anti-GFP (1:1000; Millipore), mouse anti-5-mC (1:1000; Millipore), rabbit anti-5-hmC (1:1000; Active Motif), and rabbit anti-Uhrf1 (Citterio et al. 2004). Cell death was analyzed using the Apoptag kit from Millipore according to the manufacturer's instructions.

### NSC culture

NSCs were generated from E13 *Uhrf1* floxed cortices using the protocol from Onorati et al. (2010). Electroporation was performed using the Lonza 4D nucleofector system, and cells were plated onto polyornithine- and laminin-coated dishes. *Tet2* and *Tet3* shRNA plasmids were kindly provided by Dr. Qiang Lu at the Beckman Research Institute of the City of Hope (Hahn et al. 2013). Cre-recombinant protein was added to the medium at a concentration of 14 µg/mL. The cultures were maintained for 4 d, after which they were either fixed with 4% paraformaldehyde for immunostaining analysis or used for RNA extraction.

### RNA extraction, cDNA synthesis, and qPCR

Total RNA was isolated using the Qiagen RNeasy kit for all qPCR experiments except the FACS-sorted cells. In the case of FACS-sorted cells, PicoPure RNA isolation kit from Thermo Scientific was used to extract RNA. cDNA synthesis was performed using random primers with the Maxima first strand synthesis kit (Thermo Scientific). RT-qPCR was conducted using SYBR Green and a Thermo Fisher Quant Studio 6 machine. The PCR primers used are in Supplemental Table 5.

### RNA-seq

For E16 and P5 full cortices, cerebral cortices were dissected from E16 embryos and P5 pups. Total RNA was extracted using Trizol and RNA clean and concentrator kit (Zymo Research). Ribosomal RNA was depleted with Ribozero Gold (Illumina). Libraries were prepared using NEBNext Ultra Directional RNA library preparation kit for Illumina. Quality control was carried out with a Bioanalyzer (Agilent), and 100-base-pair (bp) paired-end sequencing was performed with a HiSeq sequencer (Illumina) at Laboratory for Functional Genome Analysis (LAFUGA; Gene Center, Munich, Germany). For P5 data sets, three biological replicates were used per genotype. For E16 full cortices, two biological replicates were used for cKO, and three were used for controls.

### hmeDIP

The hmeDIP protocol was adapted from Mohn et al. (2009) and Maunakea et al. (2010). The E16 VZ was subdissected as described for the VZ RNA-seq analysis in the Supplemental Material. The antibodies used for immunoprecipitation were 5mC (Millipore) and 5hmC (Active motif). DNA libraries were generated using the NEBNext Ultra DNA library preparation kit for Illumina. Quality control was carried out with a Bioanalyzer (Agilent), and 50-bp single-end sequencing was performed with a HiSeq sequencer (Illumina) at LAFUGA.

### Ox-RRBS

Five-hundred nanograms of MspI-digested and purified DNA from controls and *Uhrf1* cKOs (E16 cortices) was used for the library preparation procedure with the NEXTflex bisulfite library preparation kit (BIOO Scientific). Library preparation was performed according to the manufacturer's instructions with some modifications. Specifically, to avoid any false positives through changes in 5hmC, a DNA oxidation step was included. Oxidation was performed with KRuO4 (Sigma-Aldrich) as described in previous work (Booth et al. 2012). Bisulfite conversion of the DNA was performed with the EZ Methylation Gold kit (Zymo Research) according to the manufacturer's instructions. Finally, quality control with a Bioanalyzer (Agilent) and sequencing with a HiSeq 1000 sequencer (Illumina, Inc.) were performed at a genomics core facility of the Center of Excellence for Fluorescent Bioanalytics (Kompetenzzentrum Fluoreszente Bioanalytik, University of Regensburg, Germany).

### Statistical analysis

For all RT-qPCR analysis, unpaired student's *t*-test was performed except in the *Tet2/3* knockdown experiments, in which a paired *t*-test was performed. For all cell counts in vivo, the Mann-Whitney test was performed. For Ox-BS analysis, Fisher's test was performed.

### Accession numbers

The accession number for the 5hmC, RNA-seq (E16 VZ, E16, and P5), and Ox-RRBS data reported in this study is GSE84550.

## Supplementary Material

Supplemental Material
