# Supplementary material for: Loss of Uhrf1 in neural stem cells leads to activation of retroviral elements and delayed neurodegeneration
Source: Genes Dev. 2016 Oct 1;30(19):2199–212. doi: 10.1101/gad.284992.116 (PMC5088568; doi:10.1101/gad.284992.116)
Supplement: Supplemental Material [file supp_30_19_2199__index.html]

Supplemental Material 

# Loss of Uhrf1 in neural stem cells leads to activation of retroviral elements and delayed neurodegeneration

## Supplemental Material

- Supplementary\_fig1.pdf
- Supplementary\_fig2.pdf
- Supplementary\_fig3.pdf
- Supplementary\_fig4.pdf
- Supplementary\_table1.pdf
- Supplementary\_text.docx
- Supplementary\_fig9.pdf
- Supplementary\_table4.pdf
- Supplementary\_fig7.pdf
- Supplementary\_fig5.pdf
- Supplementary\_table3.pdf
- Supplementary\_fig10.pdf
- Supplementary\_table5.pdf
- Supplementary\_fig8.pdf
- Supplementary\_fig6.pdf
- Supplementary\_table2.pdf
