# Supplementary figures and images for: Loss of Uhrf1 in neural stem cells leads to activation of retroviral elements and delayed neurodegeneration
Source: Genes Dev. 2016 Oct 1;30(19):2199–212. doi: 10.1101/gad.284992.116 (PMC5088568; doi:10.1101/gad.284992.116)

Adult neurogenic niches - Neural stem cells

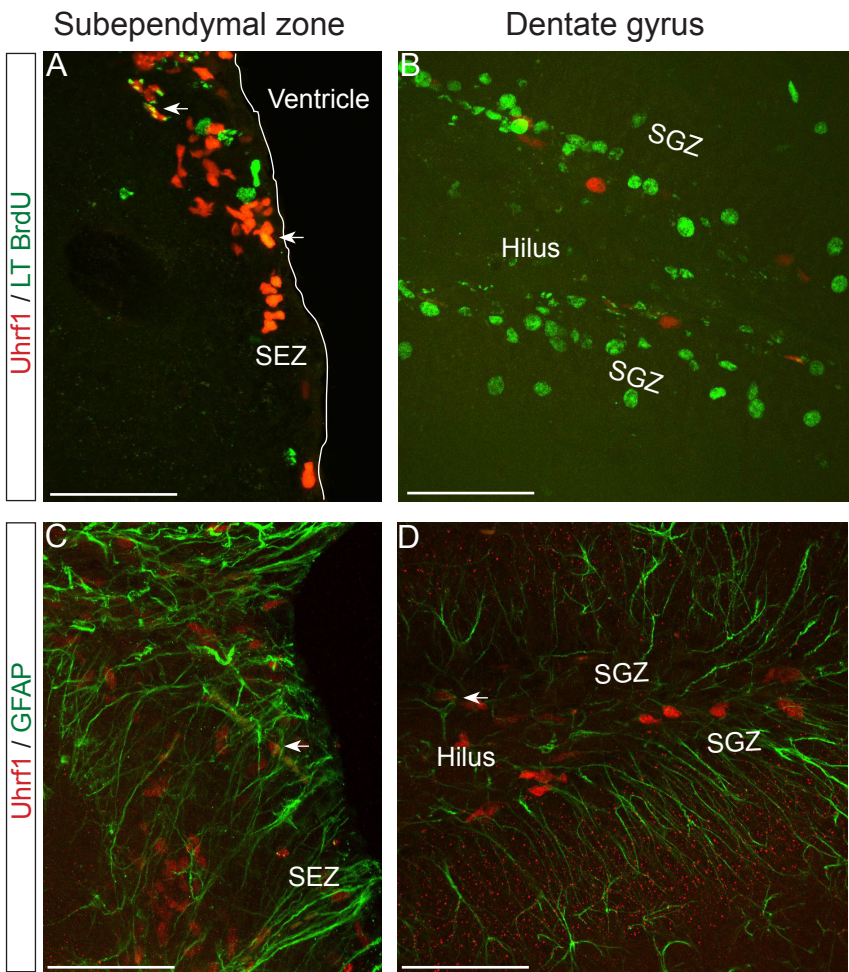

Adult neurogenic niches - Neuroblasts

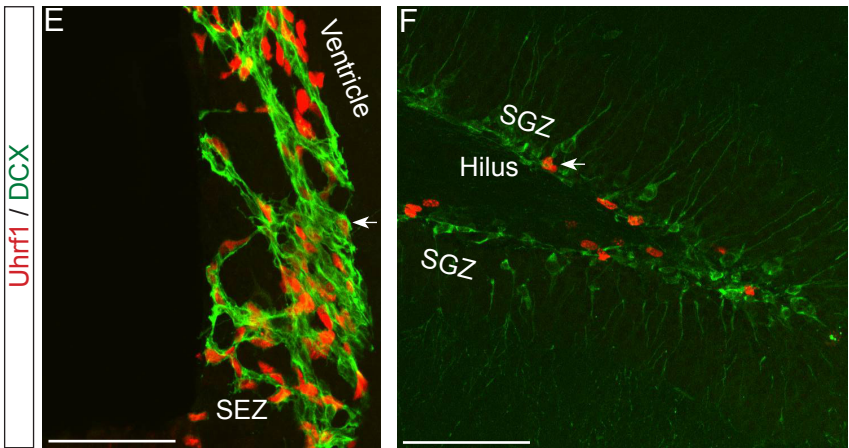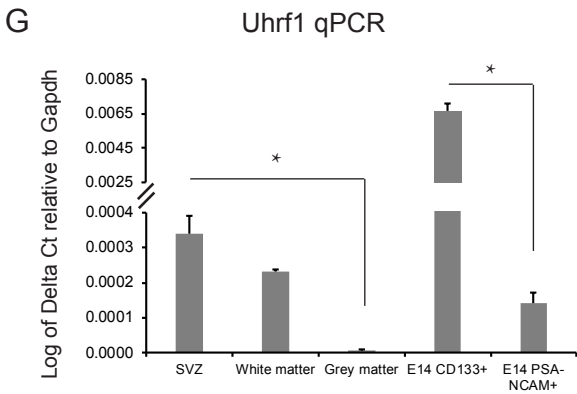

Supplement: Supplemental Material [file supp_30.19.2199_Supplementary_fig1.pdf]

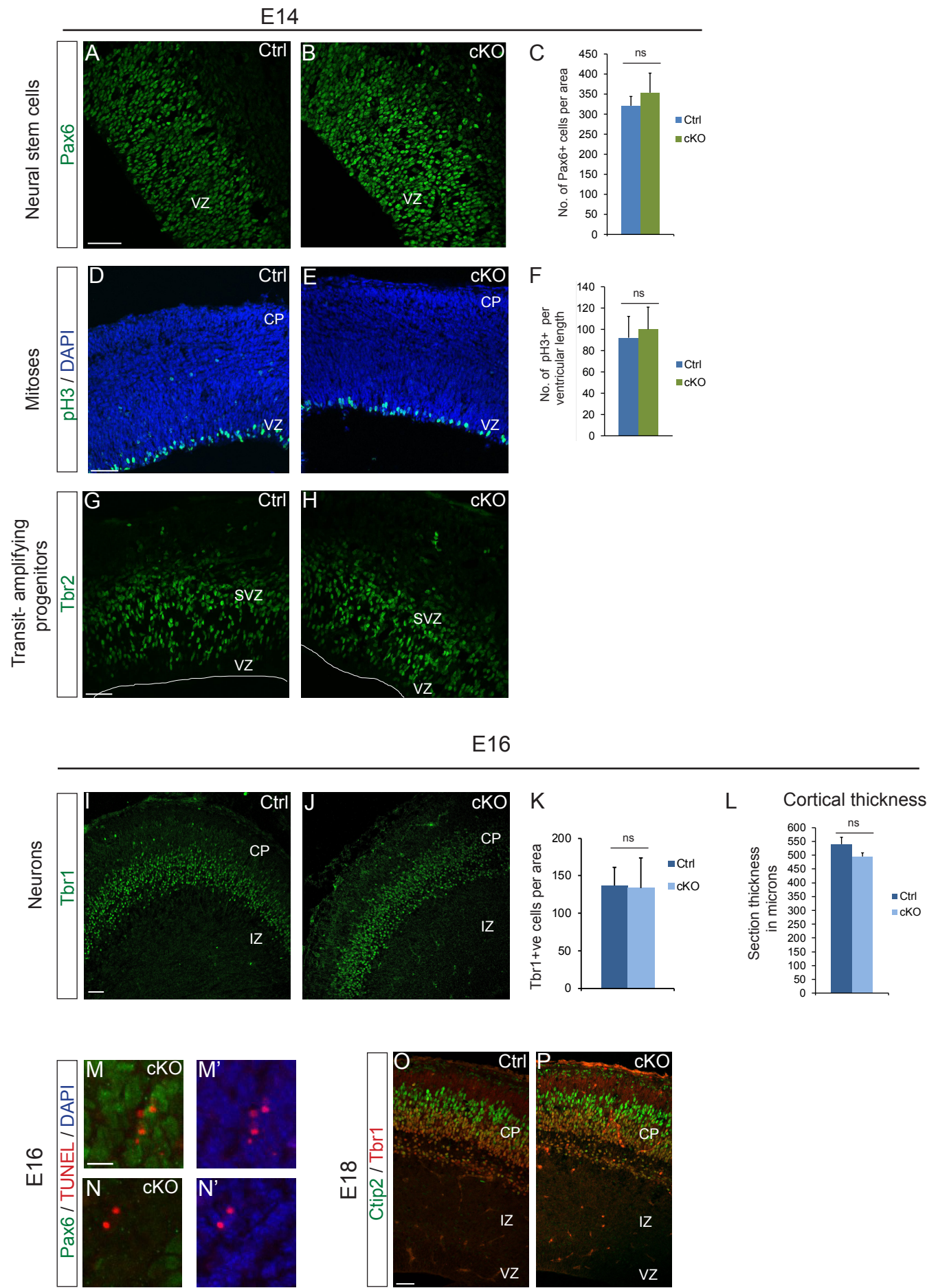

Supplement: Supplemental Material [file supp_30.19.2199_Supplementary_fig2.pdf]

P5

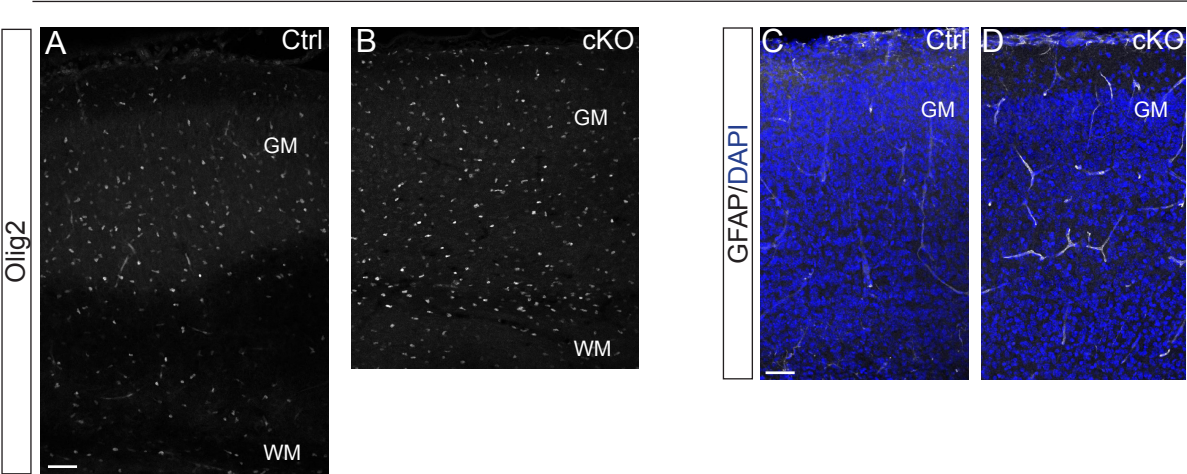

E16

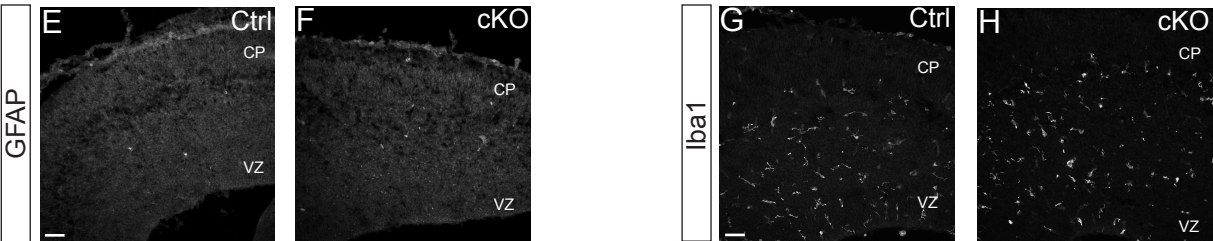

P27

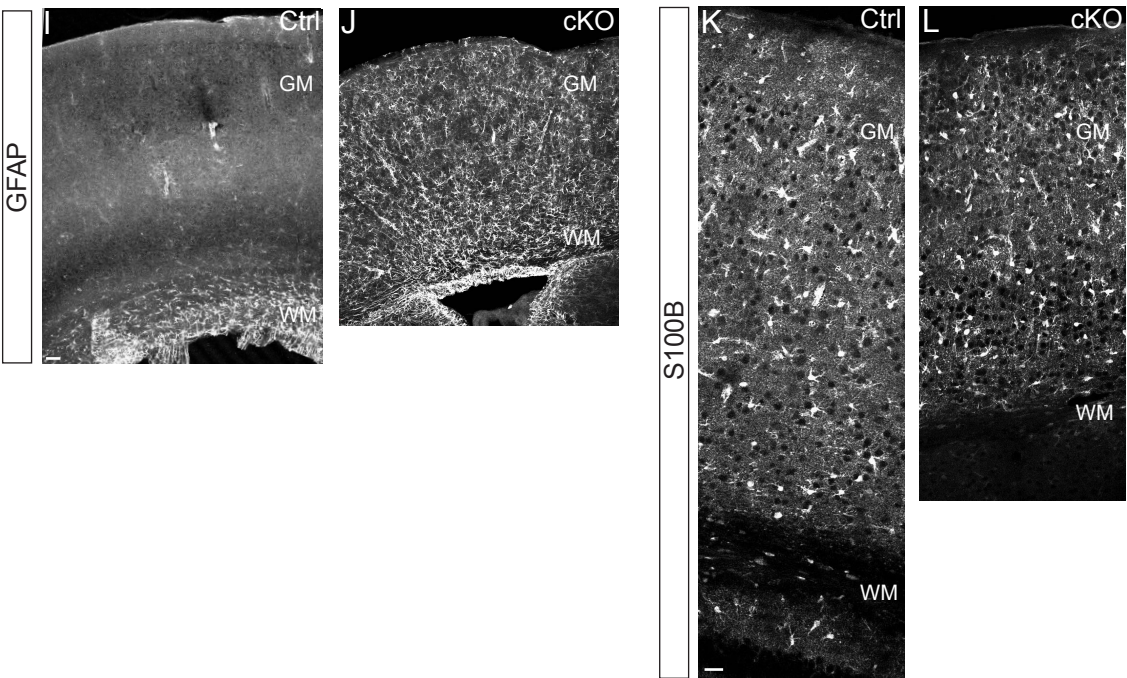

Supplement: Supplemental Material [file supp_30.19.2199_Supplementary_fig3.pdf]

A    Deregulated genes at E16 (VZ)

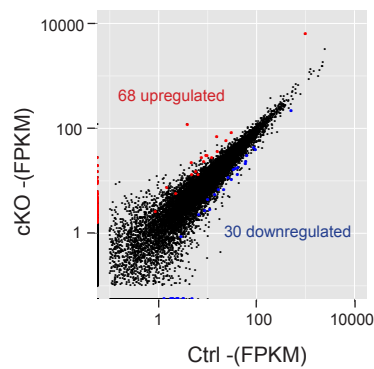

B    Gene set enrichment analysis at E16

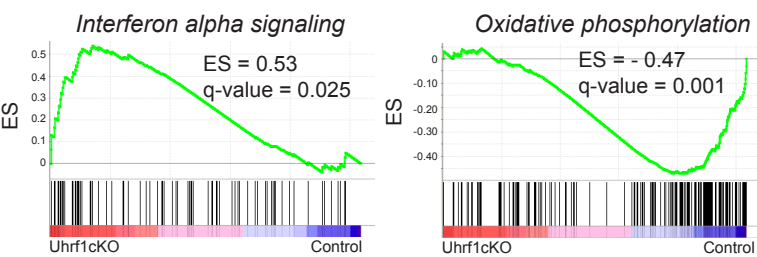

Supplement: Supplemental Material [file supp_30.19.2199_Supplementary_fig4.pdf]

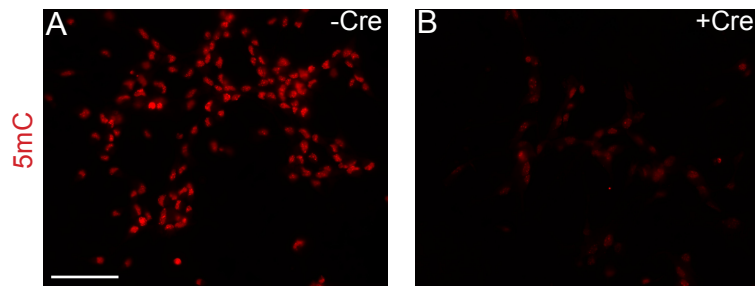

**C** NS cells - qPCR for repeat elements

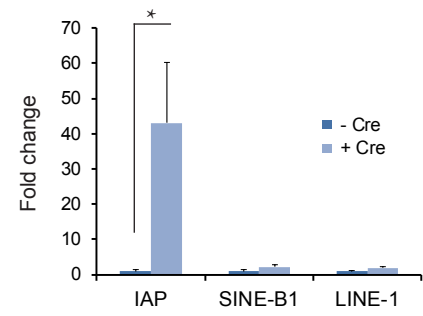

Supplement: Supplemental Material [file supp_30.19.2199_Supplementary_fig9.pdf]

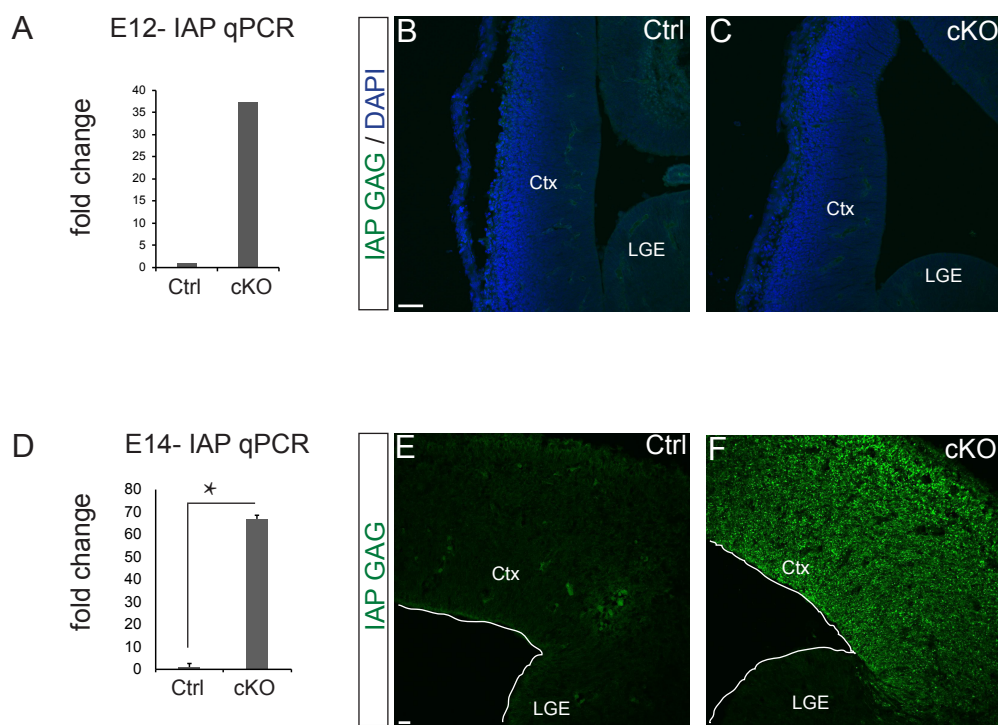

Supplement: Supplemental Material [file supp_30.19.2199_Supplementary_fig7.pdf]

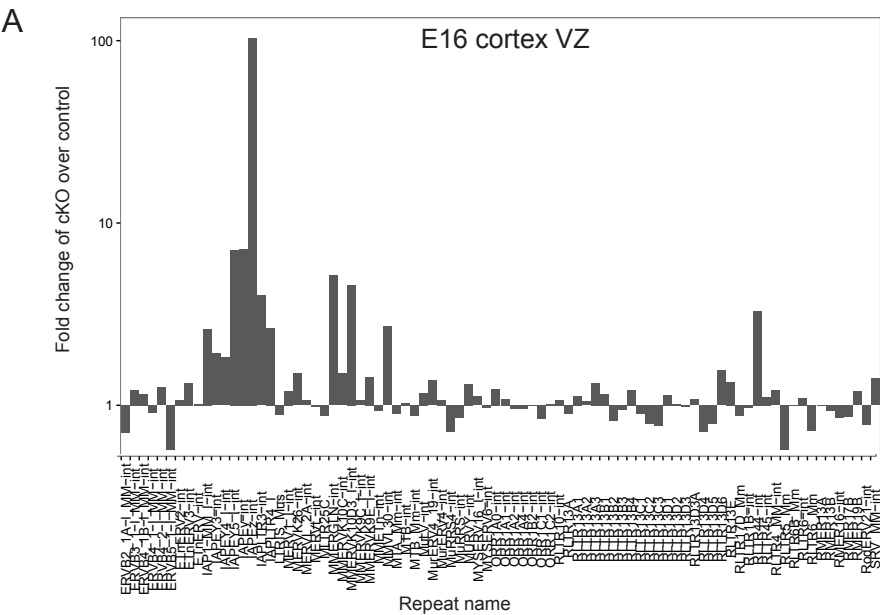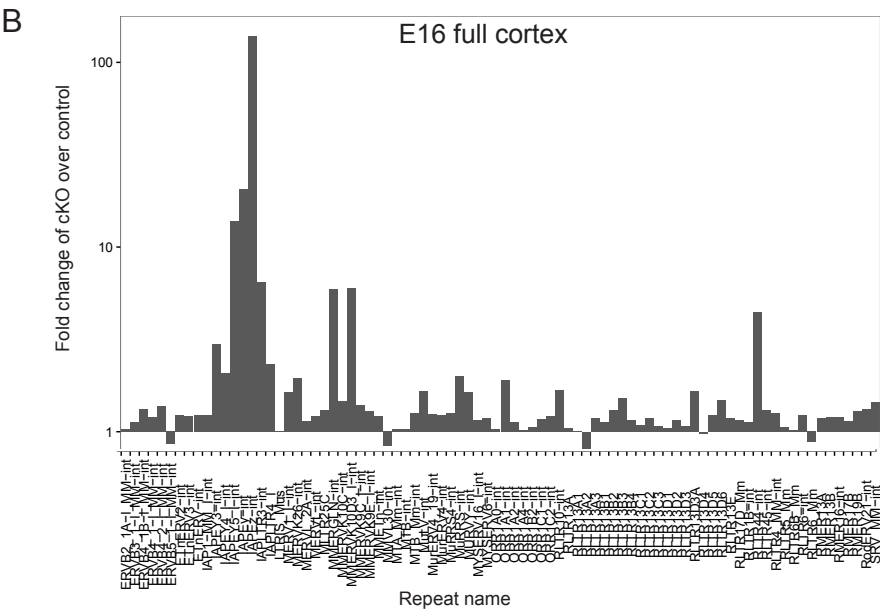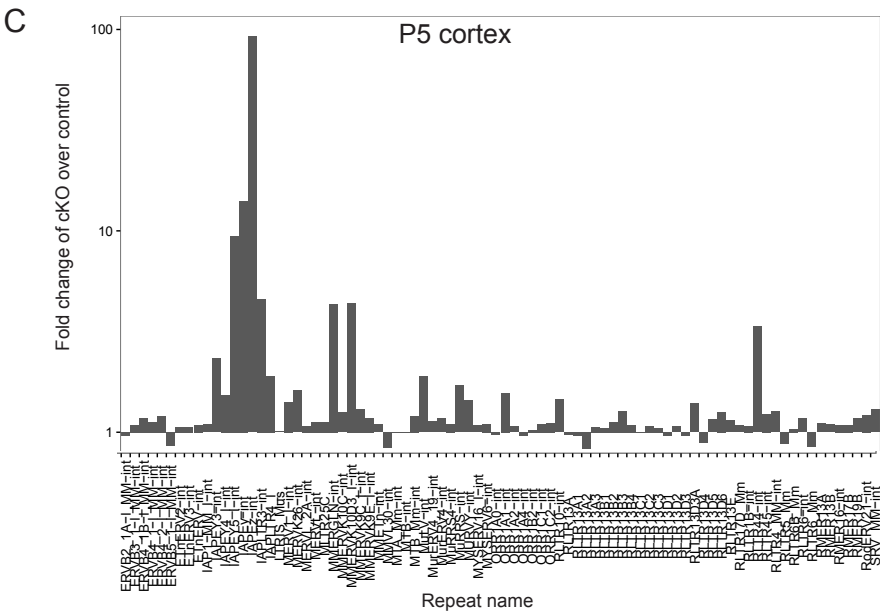

Supplement: Supplemental Material [file supp_30.19.2199_Supplementary_fig5.pdf]

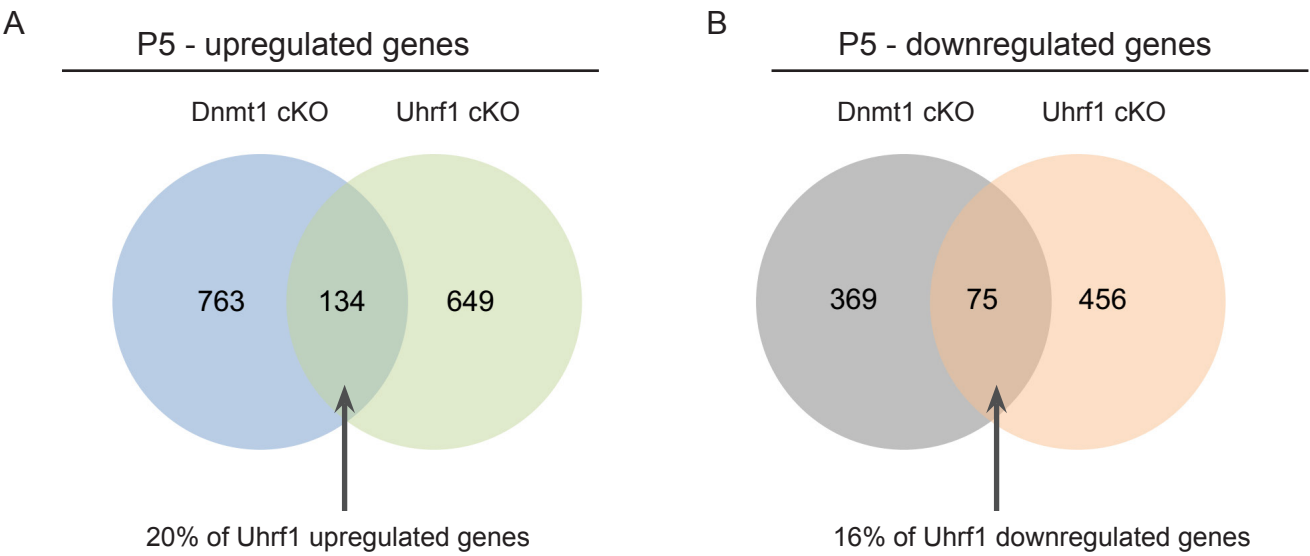

Supplement: Supplemental Material [file supp_30.19.2199_Supplementary_fig10.pdf]

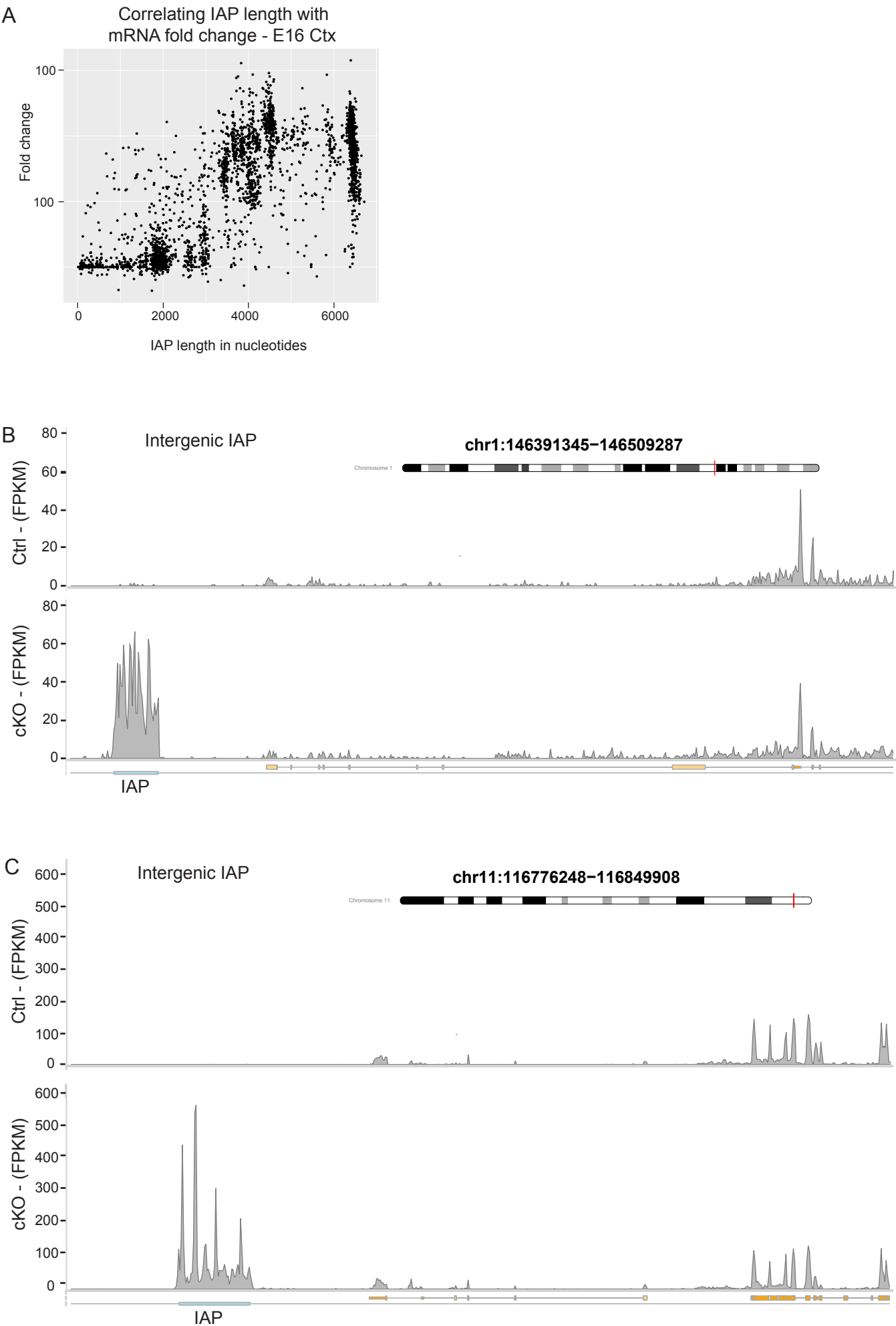

Supplement: Supplemental Material [file supp_30.19.2199_Supplementary_fig6.pdf]
