# Supplementary material for: Loss of Uhrf1 in neural stem cells leads to activation of retroviral elements and delayed neurodegeneration
Source: Genes Dev. 2016 Oct 1;30(19):2199–212. doi: 10.1101/gad.284992.116 (PMC5088568; doi:10.1101/gad.284992.116)
Supplement: Supplemental Material [file supp_30.19.2199_Supplementary_table1.pdf]

Ramesh_Supplementary table 1

RNASeq - E16

Upregulated genes						Downregulated genes		
**Gene name**	**P-value**	**FC**	**Gene name**	**P-value**	**FC**	**Gene name**	**P-value**	**FC**
Ccrn4l	5.00E-05	21.60	Pnpt1	5.00E-05	2.77	Vaultrc5	5.00E-05	0.17
Sectm1a	5.00E-05	13.54	Mbl2	5.00E-05	2.76	Lst1	5.00E-05	0.23
Mnd1	5.00E-05	10.41	Arhgap15	5.00E-05	2.72	Uhrf1	5.00E-05	0.30
AI662270	0.00015	9.97	Crlf1	5.00E-05	2.68	Hddc3	5.00E-05	0.34
Wfdc9	5.00E-05	9.84	Ulbp1	3.00E-04	2.66	Dynlt1c	5.00E-05	0.34
Trpc6	5.00E-05	9.03	Myo1a	5.00E-05	2.60	Scoc	5.00E-05	0.34
Tuba3b	5.00E-05	8.81	Zfp69	5.00E-05	2.55	Nefl	5.00E-05	0.36
Pet2	5.00E-05	7.23	Capn11	0.00035	2.55	Hbb-y	2.00E-04	0.40
Gm9866	5.00E-05	6.29	Chac1	4.00E-04	2.50	Crym	5.00E-05	0.41
4930500J02Rik	5.00E-05	5.72	Gfap	5.00E-05	2.48	Smc2os	5.00E-05	0.42
Gm773	5.00E-05	5.68	Xlr3b	5.00E-05	2.47	Gstt1	0.00105	0.42
Xlr3a	5.00E-05	5.63	Phlda3	0.00025	2.46	2610507I01Rik	5.00E-05	0.43
Slc47a1	5.00E-05	5.48	Trap1a	5.00E-05	2.43	Plin2	5.00E-05	0.48
Kntc1	5.00E-05	5.43	Ddn	5.00E-05	2.43	Gm8773	0.00065	0.48
Kcnh1	1.00E-04	5.31	Nxph4	0.00055	2.38	3110039M20Rik	5.00E-05	0.48
Uba1y	5.00E-05	4.87	Msx3	0.00015	2.36	Ublcp1	5.00E-05	0.49
Gpat2	5.00E-05	4.81	Slc1a5	5.00E-05	2.36	Tst	0.00015	0.49
Ccnb1ip1	0.00035	4.73	Cldn1	5.00E-05	2.29	Dio2	5.00E-05	0.49
Xlr4c	5.00E-05	4.72	Cilp2	5.00E-05	2.28	5730408K05Rik	6.00E-04	0.49
Hamp2	5.00E-05	4.65	Hsf2bp	1.00E-04	2.28	Morf4l1	5.00E-05	0.50
1700017N19Rik	5.00E-05	4.39	Arl10	5.00E-05	2.28			
Ctsh	5.00E-05	4.08	Rhox2d	5.00E-05	2.27			
Tspo2	5.00E-05	4.07	Relb	0.00065	2.26			
Slc7a3	5.00E-05	3.95	Ptprh	5.00E-05	2.25			
Sohlh2	5.00E-05	3.95	Ecel1	5.00E-05	2.25			
Ddr2	5.00E-05	3.93	Dmrt2	5.00E-05	2.23			
Mia	0.00105	3.90	Gramd1c	5.00E-05	2.23			
Crabp2	8.00E-04	3.87	Casq1	5.00E-05	2.23			
Cep112	5.00E-05	3.69	Tex19.1	5.00E-05	2.23			
Nxf3	5.00E-05	3.69	Cdkn1a	0.00045	2.20			
Mnd1-ps	6.00E-04	3.66	Egfr	5.00E-05	2.20			
Tfpi	5.00E-05	3.52	Ptprq	0.00105	2.20			
Eif4ebp1	5.00E-05	3.43	Dcn	1.00E-04	2.18			
Olfml2a	1.00E-04	3.41	Acrbp	1.00E-04	2.14			
Ifi27	4.00E-04	3.38	Trank1	5.00E-05	2.13			
Slc20a2	5.00E-05	3.32	Gprin3	5.00E-05	2.12			
Bfsp1	5.00E-05	3.31	Acpp	2.00E-04	2.12			
Cyct	5.00E-05	3.17	Slc6a9	0.00085	2.09			
Hspb3	0.00035	2.95	Olfr194	5.00E-05	2.08			
Tex13	5.00E-05	2.94	Stbd1	0.00015	2.08			
Cdkl4	5.00E-05	2.90	Gm5635	5.00E-05	2.07			
Nrgn	0.00015	2.84	Fam160a1	5.00E-05	2.07			
Efcab6	5.00E-05	2.80	A330102I10Rik	0.00065	2.05			
Dact2	5.00E-05	2.80	Gm6880	1.00E-04	2.04			
			Pdgfc	5.00E-05	2.02			
