# Supplementary material for: Loss of Uhrf1 in neural stem cells leads to activation of retroviral elements and delayed neurodegeneration
Source: Genes Dev. 2016 Oct 1;30(19):2199–212. doi: 10.1101/gad.284992.116 (PMC5088568; doi:10.1101/gad.284992.116)
Supplement: Supplemental Material [file supp_30.19.2199_Supplementary_text.docx]

**Ramesh et al _Supplementary data**

**Supplementary figure 1 – Uhrf1 immunostaining in adult neurogenic niches**

**A-D.** Confocal images of sagittal sections of 2-month old adult wild type brains showing the subependymal zone (**A,C,E**) and dentate gyrus (**B,D,F**) immunostained for BrdU (given for 2 weeks) and Uhrf1 to label neural stem cells in the adult neurogenic niches (**A,B**), GFAP and Uhrf1 to label NSCs (**C,D**) and doublecortin and Uhrf1 to label neuroblasts (**E,F**) as indicated in the panels.

Scale bar = 50µm; SEZ – subependymal zone; SGZ – subgranular zone

**G.** qPCR for Uhrf1 in embryonic NSCs and neurons and adult SVZ, white matter and Grey matter. The graph shows the almost absence of Uhrf1 mRNA in adult grey matter containing mainly neurons. Error bars indicate standard error of mean. n=3. * indicates significance with p-value=0.009 and 0.00005 respectively.

**Supplementary figure 2 – Neurogenesis during embryonic development is largely unaffected by Uhrf1 deletion in the cerebral cortex**

Confocal images of coronal sections of E14 and E16 control (**A,D,G,I**) and cKO (**B,E,H,J**) cerebral cortex, stained for Pax6 to label neural stem cells in the ventricular zone (**A-C**), pH3 to detect cells in mitosis (**D-F**), Tbr2 to label basal progenitors in the subventricular zone (**G,H**), and Tbr1 to label neurons (**I-K**). Note that all these cells types are located at their normal position and present in the same number in control or cKO cerebral cortex (for quantification see **C,F** and **K,L**). ns indicates no significance with p-value = 0.6 for **C** and **F** and p-value = 0.2 for **K** and **L** graphs.

**M,N.** Confocal images of coronal sections of E16 cKO cerebral cortex stained for TUNEL and Pax6, indicating both Pax6 positive and negative/TUNEL positive cells.

**O,P.** Confocal images of coronal sections of E18 control and cKO cerebral cortex stained for Ctip2 and Tbr1, indicating comparable cortical layering between controls and Uhrf1 cKO.

Error bars indicate standard error of mean; n=4 or 5.

Scale bar indicates 10 µm for panels **M,N** only and 50 µm for all other panels.

VZ – ventricular zone; SVZ –subventricular zone; CP – cortical plate; IZ – intermediate zone

**Supplementary figure 3 – Gliogenesis proceeds normally after Uhrf1 deletion in the cerebral cortex**

**A-D.** Confocal images of coronal sections of P5 cerebral cortex of controls (**A, C**) and Uhrf1 cKO (**B,D**) immunostained for Olig2 to label oligodendrocyte progenitors (**A,B**) and GFAP to label astrocytes (**C,D**). Note that Olig2+ cells are present in equal density and GFAP is still largely absent both in control and cKO P5 cerebral cortex.

**E-H.** Confocal images of coronal sections of E16 cerebral cortex of controls (**E,G**) and Uhrf1 cKO (**F,H**) immunostained for GFAP (**E,F**) and Iba1 to label microglia (**G,H**) Note that GFAP is still absent both in control and cKO E16 cerebral cortex and microglia look comparable in number and morphology.

**I-L.** Confocal images of coronal sections of P27 cerebral cortex of controls (**I,K**) and Uhrf1 cKO (**J,L**) immunostained for GFAP to label astrocytes in the white matter (**I,J**) and reactive astrocytes (**J**) and S100B to label all astrocytes (**K,L**). Note that the astrocyte density is not altered in the cKO compared to control cerebral cortex (**K,L**), but astrocytes in the cKO grey matter strongly upregulate GFAP (**J**).

Scale bar = 50µm; WM- white matter; GM- grey matter; CP- cortical plate; VZ – ventricular zone

**Supplementary figure 4 – Activation of IAP retroviral elements in Uhrf1cKO cerebral cortex ventricular zones**

**A.** Graph depicting deregulated genes in E16 cKO ventricular zone tissue. Red dots indicate genes with significantly increased expression and blue dots indicate genes with significantly downregulated expression in the cKO cerebral cortex compared to controls. Black dots are genes with unchanged expression level. Significantly regulated genes have p-value less than 0.05 and fold change above 2. X axis and Y axis values are FPKM (Fragments per million bases) from 100bp paired end RNAseq.

**B.** Graphs showing gene set enrichment analysis of Hallmark datasets, from the RNAseq analysis from **A**. The graph depicts an overrepresentation of genes related to interferon alpha signalling and underrepresentation of genes related to oxidative phosphorylation in the cKO. ES- Enrichment score. FDR < 25%.

**Supplementary figure 5** – **Bar graphs showing upregulated ERV repeat classes**

Upregulated ERV repeat classes based on Repbase classification in E16 VZ RNAseq (**A**), E16 full cortex RNAseq (**B**) and P5 RNAseq (**C**). Y-axis is fold change and X-axis is each repeat class. Note that IAP classes are the most upregulated in all datasets.

**Supplementary figure 6 – Intergenic and long IAP upregulation in P5 RNAseq**

**A.** Graph plotting length of IAP sequence against RNAseq fold change, indicating a bias for long IAP transcripts to be activated.

**B-C.** Snapshots of IGV browser showing intergenic IAP upregulation in Uhrf1 ckO. Y-axis is RPKM values. X-axis is a magnification of the region on the chromosome marked in red containing the genes and IAP in proximity.

**Supplementary figure 7– IAP activation in Uhrf1 cKO cerebral cortex occurs at early embryonic stages**

**A,D.** RT-qPCR graph for IAP in E12 (**A**) and E14 (**D**) control and cKO cerebral cortex tissue. * indicates significance with p-value = 0.004. Error bars indicates standard error of mean. n=3 for E14 and n=2 for E12.

**B-C, E-F.** Confocal images of coronal sections of E12 (**B,C**) and E14 (**E,F**) cerebral cortex of controls (**C,E**) and cKO (**D,F**) immunostained for IAP GAG antibody and DAPI as indicated in the panels.

Scale bar = 50µm; LGE – Lateral Ganglionic Eminence; Cortex = cerebral cortex.

**Supplementary figure 8 – Correlation between changes in methylation and mRNA expression in E16 cKO cerebral cortex**

**A.** Venn diagram depicting the few overlapping genes that are upregulated in expression and hypomethylated in E16 Uhrf1 cKO cerebral cortex. For the E16 upregulated genes, the cutoff is p value < 0.05 with fold change > 1.5. For hypomethylated promoters, q-value < 0.01 and atleast 25% loss of 5mC.

**B.** Graph plotting fold change of LINE 1 elements from RNAseq against Ox-RRBS data for 5mc levels, showing few hypomethyated elements with high fold change in mRNA. Red dots indicate LINE-1 elements in proximity to IAP elements. Black dots are all remaining LINE-1 elements.

**C.** Graph showing hypomethylation of LINE 1 regulatory regions of two LINE-1 repbase classes.

**D**. Table depicting IAP classes with high 5hmC (> 2 fold change). All classes were observed to have increase mRNA levels on the E16 RNAseq datasets.

**Supplementary Figure 9 – NS in vitro system shows hypomethylation on Cre induction and specificity for IAP transcriptional regulation**

**A,B**. Uhrf1 floxed NS cells immunstained for 5mC with and without Cre induction, indicating loss of 5mC on Uhrf1 deletion, after 4 days in vitro.

**C**. qPCR for retrotransposons with and without Cre induction in Uhrf1 floxed NS cells, after 4 days in vitro. Graph indicates that only IAP is specifically upregulated.

* indicates significance with p value = 0.001; n=4; Error bars indicate standard error of mean.

**Supplementary figure 10– Comparison of gene regulation upon Uhrf1 or Dnmt1 deletion with Emx1-driven Cre deletion**

**A, B.** Venn diagrams depicting genes with upregulated (**A**) or down-regulated (**B**) expression levels in Uhrf1 and (overlap)/or (separate parts in the pies) DNMT1 cKO cerebral cortex at postnatal day 5. Note that in both cKOs, more genes have increased expression levels, but only 20% (A) or 16% (B) of the genes changed in expression in the Uhrf1 cKO are also regulated in the DNMT1 cKO cerebral cortex, indicating that many effects of Uhrf1 deletion are not shared with DNMT1 deletion.

For the p5 deregulated genes, the cutoff is p value < 0.05 with fold change > 1.5.

**Supplementary table 1 – Genes up- or down-regulated in expression at E16 in Uhrf1 cKO cerebral cortex.**Table depicting genes with significantly increased and decreased expression levels in the E16 Uhrf1 cKO cerebral cortex .

Green indicates genes related to testis/germ cell and orange indicates derepressed genes.

**Supplementary table 2 – Genes up- or down-regulated in expression at P5 in Uhrf1 cKO cerebral cortex.**Table depicting genes with significantly increased and decreased expression levels in the E16 Uhrf1 cKO cerebral cortex .

**Supplementary table 3 – Deregulated genes in proximity to deregulated IAP transcripts**Table depicting percentage and list of genes in proximity to IAPs, upregulated in Uhrf1 cKO. as – antisense; s – sense; i – intergenic; u – upstream from gene

**Supplementary table 4 - Genes upregulated in expression at P5 in both Uhrf1 and Dnmt1 cKO cerebral cortex.**

Table depicting genes with expression upregulated in both Uhrf1 and Dnmt1 cKO cerebral cortex. Red indicates genes which are hypomethylated in the E16 cortex of Uhrf1 cKO.

**Supplementary table 5 – Primer List**

List of primers used in this study

**Supplementary methods**

**Methyl sensitive restriction digests**

Genomic DNA was isolated from E16 controls and Uhrf1 cKO cortices and digested with either HpaII or MspI using the EpiJET DNA methylation analysis kit from Thermofisher Scientific according to manufacturer’s instructions.

**RNAseq - E16 ventricular zone**

Entire cortices were dissected from E16 embryos and sectioned with the tissue chopper at 300µm thickness. Slices were cut into half with the lower part being considered as the germinal zones. Total RNA was extracted using trizol and RNA clean and concentrator kit (Zymo Research). Ribosomal RNA was depleted with Ribogone mammalian kit from Clontech. Libraries were prepared using SMARTer Stranded RNA-Seq Kit from Clontech. Quality control was carried out with a Bioanalyzer (Agilent) and 100bp paired end sequencing with a HiSeq sequencer (Illumina) at LAFUGA. For E16 VZ datasets, 3 biological replicates were used per genotype.

**In utero electroporation**

Animals were operated as approved by the Government of Upper Bavaria under licence number 55.2-1-54-2532-79/11. E13.5 timed pregnant mice were anaesthetized by intra-peritoneal (i.p.) injection of Fentanyl (0.05mg/kg), Midazolam (5mg/kg) and Medetomidine (0.5mg/kg) (Btm license number: 4518395). Tet2 and Tet3 shRNA plasmid were kindly provided by Professor Qiang Lu at the Beckmann research institute, USA (Hahn et al. 2013) . Plasmids were mixed with Fast Green (2.5mg/μl, Sigma) and injected at a concentration of 1 µg/µl. In utero electroporations were done as described earlier (Saito, 2006). Anesthesia was terminated by subcutaneous injection of Buprenorphine (0.1mg/kg), Atipamezol (2.5mg/kg), Flumazenil (0.5mg/kg). Embryonic brains were fixed and sliced as described in the main methods. Embryos were fixed and analysed as mentioned in the main methods.

**Fluoroscence Activated Cell Sorting**

Cortices from E16 embryos were isolated and the midline and meninges removed. The tissue was enzymatically dissociated using 0.05% Trypsin and resuspended in DMEM containing 10% fetal calf serum (FCS) then washed with PBS. For isolation of GFP+ cells (after in utero electroporation), the positive gate was set using non-electroporated tissue. The positive gates were set to include a maximum 0.1% of non-fluorescent cells. Positive cells were isolated using the FACSAriaIII (BD Bioscience) system at purity mode. Cells were directly sorted into PBS.

For isolation of cortical subpopulations (neural stem cells and neurons) the samples were incubated with APC-conjugated Prominin-1 antibody (CD133, eBioscience) and PE-conjugated PSA-NCAM antibody (Miltenyi Biotec). Gating parameters were determined by side and forward scatter to eliminate debris (P1 gate) and aggregated cells (P2 gate).PE- and APC conjugated isotype control (eBioscience, Miltenyi Biotec) stained cells were used to set the positive gates for the stained populations.

**Oxidative Reduced Representation Bisulfite Sequencing**

1 µg of mouse DNA from E16 control and Uhrf1 cKOs was digested with MSPI (NEB) in Buffer 4 at 37°C O/N. The digested DNA was cleaned up with the MinElute Reaction Cleanup Kit (Qiagen) according to the manufacturer´s instructions. 500 ng of digested and purified DNA was used for the library preparation procedure with the NEXTflex™ Bisulfite Library Prep Kit (BIOO Scientific). Library preparation was performed according to the manufacturer´s instructions with some modifications. Specifically, to avoid any false positives through changes in 5hmC, a DNA oxidation step was included. The oxidation step converts 5hmC to 5fmC but does not affect 5mC. Oxidation was performed with KRuO4 (Sigma-Aldrich) as described in previous work (Booth et al. 2012). Bisulfite conversion of the DNA was performed with the EZ Methylation Gold Kit (Zymo Research) according to the manufacturer´s instructions. The libraries were purified using AMPure XP beads (Beckmann Coulter) and PCR amplification of the oxidized and bisulfite converted libraries was performed with PfuCx Hot Start (Agilent). Finally, quality control with a Bioanalyzer (Agilent) and sequencing with a HiSeq 1000 sequencer (Illumina Inc.) was performed at a genomics core facility: Center of Excellence for Fluorescent Bioanalytics (KFB, University of Regensburg, Germany.

**Oxidative Bisulfite sequencing**

gDNA was extracted from E16 cortical tissue from controls and Uhrf1 cKO and Uhrf1 floxed NS cells with and without cre. Oxidation and bisulfite conversion was performed as described in the Ox-RRBS section in the main methods. The IAP GAG region and LINE-1 was amplified by PCR (list of primers in Supplementary table 5). Primary amplicons were adapter tagged using NEBNext Multiplex Oligos for Illumina (New England Biolabs). 100-200 ng of amplicon and 62.5 pmol each of universal PCR primer and index primer were amplified in a total volume of 50 µL with NEBNext High-Fidelity PCR Master Mix (New England Biolabs) and the cycling conditions 98°C/30"-[98°C/10"-65°C/30"-72°C/30"]x4-72°C/5'. PCR reactions were purified adding 80% (v/v) CleanPCR magnetic beads (GC biotech) and finally eluted with 25 µL water. A single round of amplification with P5 and P7 primers (Illumina) was added to make all fragments double stranded. For this, 20 µL of the purified tagged amplicons and 25 pmol each of P5 and P7 primers were set up in a total volume of 50 µL with Phusion Polymerase (New England Biolabs) according to the manufacturer's instructions, and cycled with the program 98°C/2'-60°C/1'-72°C/10'. Purification was the same as before. Libraries were checked for correct fragment length on a Bioanalyzer 2100 (Agilent) and concentrations were determined with Qubit dsDNA HS Assay Kit (Life Technologies). High-Throughput Sequencing (2x300 bp paired end) was performed on a MiSeq sequencer with v3 chemistry (Illumina) at the Genomics Service Unit at LMU Biocenter. Primary sequencing reads were merged using CLC Genomics Workbench 9.0.1 (Qiagen) with the following parameters: mismatch cost = 2; minimum score = 8; gap cost = 3; maximum unaligned end mismatches = 0.

**Hydroxy methylated DNA immunoprecipitation (hmeDIP)**

The protocol was adapted from chapter 5 of book ‘DNA methylation:Methods and Protocols, second edition volume 507, 2009 – Fabio Mohn, Micheal Weber, Dirk Schubeler and Tim-Christoph Roloff and from (Maunakea et al. 2010). The E16 ventricular zone was subdissected as described for the VZ RNAseq analysis in supplementary material. DNA was lysed in Tris-EDTA buffer with 0.5% SDS and extracted by phenol-chloroform extraction. Chromatin was cheared using the Covaris machine and 1ug of gDNA was used per immunoprecipitation (IP). The supernatant from the IP was purified with Qiagen PCR purification kit. The antibodies used for immunoprecipitation were 5mC (Millipore) and 5hmC (Active motif). DNA libraries were generated using the NEBNext Ultra DNA library preparation kit for Illumina. Quality control was carried out with a Bioanalyzer (Agilent) and 50bp single end sequencing with a HiSeq sequencer (Illumina) at LAFUGA.

**Bioinformatic analyses**

***RNA-seq***

RNA-seq reads (100bp paired-end) were mapped to the mouse genome (mm10) using tophat2 (Kim et al. 2013), resulting in alignments with minimal mismatches. The resulting BAM files were filtered to only retain reads in a proper pair and to remove reads with multiple mapping in the genome as well as duplicated reads and low quality alignments using samtools (-F 2 -f 1280)*.* Expression of genes in FPKM was calculated with cuffdiff and cummerbund (Trapnell et al. 2013).Up- and down-regulated genes were selected based on the cufflinks p-value (p<0.05) and change in expression (> 2 fold). De-repressed genes were selected based on very low expression in control (FPKM < 0.1) and higher expression in cKO (FPKM > 0.1). Dot plots were generated with ggplot2. Gene set enrichment analysis of differentially expressed genes was performed as described using the HALLMARKS gene set (Mootha et al. 2003; Subramanian et al. 2005).

For expression analysis of individual repeat elements, filtered BAM files were converted to homer tag directories using makeTagDirectory. Genome positions for individual IAP elements were extracted from the rmsk dataset (UCSC) and coverage across repeat families and individual repeats was analyzed using analyzeRepeats (Heinz et al. 2010).

Genes which may be regulated by derepressed IAP elements were identified by (1) selecting upregulated genes (fold change > 2); (2) testing for activated IAP elements within upregulated genes (+/- 50.000 bp).

***Hydroxy-meDIP***

Hydroxy-MeDIP reads (50bp single end) were mapped to the mouse genome (mm10) using bowtie with options -q –n 2 --best. Resulting SAM files were converted to homer tag directories using makeTagDirectory (Heinz et al. 2010). Cumulative IAP coverage plots were generated as in (Sadic et al. 2015).

***Oxidative Reduced Representation Bisulfite Sequencing***

All reads generated were mapped to the mouse genome version GRCm38 with the commercially available Genomatix software (version 2.2.2 Genomatix) allowing no mismatch. For analysis of the control sequences and the cKO status, a custom mapping library with the methylated and unmethylated DNA sequences of the PCR products and vector, respectively, was generated. From these mappings, the methylation status per CpG or CpH was extracted, positions with a coverage = < 5 were excluded and further data analysis was performed with custom scripts using the free software R ver. 3.1.1 and the package methylkit (Akalin et al. 2012). For the analysis of differentially methylated regions, the region length was set to 100 bp.

Genomatix was used to predict promoter regions that were used for differential methylation analysis.

***Oxidative Bisulfite sequencing***

All reads generated were filtered for size (490bp to 510bp for IAP and 250-270bp for LINE-1) using Perl. 300 independent PCR products were subsampled per condition and analysed with QUMA for their methylation status per CpG. % of 5hmC was inferred from the difference between oxidized and unoxidised samples, per condition.

**Supplementary references**

Akalin A, Kormaksson M, Li S, Garrett-Bakelman FE, Figueroa ME, Melnick A, Mason CE. 2012. methylKit: a comprehensive R package for the analysis of genome-wide DNA methylation profiles. *Genome Biol* **13**: R87.

Booth MJ, Branco MR, Ficz G, Oxley D, Krueger F, Reik W, Balasubramanian S. 2012. Quantitative sequencing of 5-methylcytosine and 5-hydroxymethylcytosine at single-base resolution. *Science* **336**: 934-937.

Hahn MA, Qiu R, Wu X, Li AX, Zhang H, Wang J, Jui J, Jin SG, Jiang Y, Pfeifer GP et al. 2013. Dynamics of 5-hydroxymethylcytosine and chromatin marks in Mammalian neurogenesis. *Cell Rep* **3**: 291-300.

Heinz S, Benner C, Spann N, Bertolino E, Lin YC, Laslo P, Cheng JX, Murre C, Singh H, Glass CK. 2010. Simple combinations of lineage-determining transcription factors prime cis-regulatory elements required for macrophage and B cell identities. *Mol Cell* **38**: 576-589.

Kim D, Pertea G, Trapnell C, Pimentel H, Kelley R, Salzberg SL. 2013. TopHat2: accurate alignment of transcriptomes in the presence of insertions, deletions and gene fusions. *Genome Biol* **14**: R36.

Maunakea AK, Nagarajan RP, Bilenky M, Ballinger TJ, D'Souza C, Fouse SD, Johnson BE, Hong C, Nielsen C, Zhao Y et al. 2010. Conserved role of intragenic DNA methylation in regulating alternative promoters. *Nature* **466**: 253-257.

Mootha VK, Lindgren CM, Eriksson KF, Subramanian A, Sihag S, Lehar J, Puigserver P, Carlsson E, Ridderstrale M, Laurila E et al. 2003. PGC-1alpha-responsive genes involved in oxidative phosphorylation are coordinately downregulated in human diabetes. *Nat Genet* **34**: 267-273.

Sadic D, Schmidt K, Groh S, Kondofersky I, Ellwart J, Fuchs C, Theis FJ, Schotta G. 2015. Atrx promotes heterochromatin formation at retrotransposons. *EMBO Rep* **16**: 836-850.

Subramanian A, Tamayo P, Mootha VK, Mukherjee S, Ebert BL, Gillette MA, Paulovich A, Pomeroy SL, Golub TR, Lander ES et al. 2005. Gene set enrichment analysis: a knowledge-based approach for interpreting genome-wide expression profiles. *Proc Natl Acad Sci U S A* **102**: 15545-15550.

Trapnell C, Hendrickson DG, Sauvageau M, Goff L, Rinn JL, Pachter L. 2013. Differential analysis of gene regulation at transcript resolution with RNA-seq. *Nat Biotechnol* **31**: 46-53.

Jörg Tost (ed.), DNA Methylation: Methods and Protocols, Second Edition, vol. 5072009 Humana Press, a part of Springer Science+Business Media DOI 10.1007/978-1-59745-522-0 5 Springerprotocols.com
