# Supplementary material for: Loss of Uhrf1 in neural stem cells leads to activation of retroviral elements and delayed neurodegeneration
Source: Genes Dev. 2016 Oct 1;30(19):2199–212. doi: 10.1101/gad.284992.116 (PMC5088568; doi:10.1101/gad.284992.116)
Supplement: Supplemental Material [file supp_30.19.2199_Supplementary_table4.pdf]

Genes upregulated in both Dnmt1 and Uhrf1 cKO at P5

1700018B24Rik	Ddr2	Lgals3bp	Rbp1
1700029P11Rik	Dlx2	Lhx6	Rest
1700080O16Rik	Dpep1	Lin28b	Rhbdl3
4930500J02Rik	Eda2r	Madcam1	Rhox5
6720468P15Rik	Egfr	Mbl2	Ripk4
AI662270	Eif4ebp1	Meis1	S100b
Adamts19	Fos	Mitf	Saa3
Adarb2	Gad1	Mpeg1	Sema3b
Apobec1	Gad2	Ms4a6d	Serpinb1a
Apoc1	Gbp2	Msx1	Serpine1
Atf3	Gem	Mthfd2	Sesn2
Atf5	Gm9	Mypn	Sirt5
Avil	Gpc3	Nat2	Slc7a3
C1qc	Gpnmb	Neb	Spp1
Car13	Gramd1c	Ngfr	Svs5
Cbx7	Grhl2	Nupr1	Tal1
Ccnd2	Grik1	Nxph1	Tcfl5
Cd74	H19	Oas2	Tdrd9
Cd84	H2-Ab1	Olfml2a	Tex101
Cdkn2b	H2-M3	Olfr1347	Tfpi
Chac1	Hamp2	Pard6b	Tlr2
Cldn11	Hfe	Parp12	Tmed2
Clic1	Hk2	Parp14	Trem2
Clic4	Hmx1	Parp3	Trim66
Col1a1	Id1	Pdlim4	Trp53inp1
Cox6a2	Ier3	Pet2	Ttn
Cox8c	Ifitm3	Pgam2	Tyrobp
Crb1	Igf1	Pipox	Ugt3a2
Creb3l2	Igfbpl1	Piwil2	Vim
Ctss	Il15	Pnpt1	Wfdc12
Cxcr4	Inadl	Psma8	Xlr3b
Cyb5r1	Irf1	Psph	Zfp536
Cyp1b1	Kcnc2	Rab39	
Cypt12	Kntc1	Rabggta	
