# Supplementary material for: Loss of Uhrf1 in neural stem cells leads to activation of retroviral elements and delayed neurodegeneration
Source: Genes Dev. 2016 Oct 1;30(19):2199–212. doi: 10.1101/gad.284992.116 (PMC5088568; doi:10.1101/gad.284992.116)
Supplement: Supplemental Material [file supp_30.19.2199_Supplementary_table3.pdf]

Ramesh_Supplementary table 3

Correlation between deregulated IAP transcripts and proximal genes

Deregulated genes neighbouring deregulated IAPs

	% of deregulated genes
E16 VZ	16
E16 full cortex	19
P5	33

E16 RNAseq

Gene name	IAP
Ccrn4l	,i.s
Sectm1a	,i.as
Mnd1	,i.s
Trpc6	,i.s
Slc47a1	,u.as
Kcnh1	,u.s
Hamp2	,u.s
1700017N19Rik	,u.s
Ddr2	,u.as
Cep112	,i.s,u.s
Tfpi	,u.as
Slc20a2	,i.s
Efcab6	,i.as,u.as
Pnpt1	,u.s
Ulbp1	,d.as
Zfp69	,i.s
Capn11	,i.as
Ecel1	,d.s
Gramd1c	,u.s
Casq1	,u.as
Olfr194	,u.s
Fam160a1	,d.s
Arhgef18	,u.s

P5 RNAseq

Gene name	IAP	Gene name	IAP
2010005H15Rik	,d.as	Mnd1	,i.s
Cypt12	,d.as	Mrgprx2	,i.s
Hcst	,d.as	Slc20a2	,i.s
Klhl33	,d.as	Trpc6	,i.s
Mypn	,d.as	Zfp69	,i.s
Trim34a	,d.as	Cep112	,i.s,u.s
Ulbp1	,d.as	Mitf	,i.s,u.s
Cldn11	,d.s	Casq1	,u.as
D630041G03Rik	,d.s	Ddr2	,u.as
Ecel1	,d.s	Eif2s3y	,u.as
Fam160a1	,d.s	Foxd1	,u.as
Olfr498	,d.s	Slc47a1	,u.as
Psmb8	,d.s	Tfpi	,u.as
Scel	,d.s	1700017N19Rik	,u.s
Tap1	,d.s	1700080O16Rik	,u.s
Tap2	,d.s	Arhgef18	,u.s
Apoh	,i.as	BC035044	,u.s
Capn11	,i.as	Dhrs3	,u.s
Cped1	,i.as	E330017A01Rik	,u.s
Enpp1	,i.as	Gramd1c	,u.s
F13a1	,i.as	Gsdma3	,u.s
Mageb18	,i.as	H2-Ab1	,u.s
Mei4	,i.as	H2-Q4	,u.s
Sectm1a	,i.as	Hamp2	,u.s
Efcab6	,i.as,u.as	Ikzf2	,u.s
A330049N07Rik	,i.s	Kcnh1	,u.s
Ccrn4l	,i.s	Olfr1054	,u.s
Cdh19	,i.s	Olfr193	,u.s
Itgbl1	,i.s	Olfr194	,u.s
Mmp1b	,i.s	Olfr43	,u.s
		Pnpt1	,u.s
