# Supplementary material for: Loss of Uhrf1 in neural stem cells leads to activation of retroviral elements and delayed neurodegeneration
Source: Genes Dev. 2016 Oct 1;30(19):2199–212. doi: 10.1101/gad.284992.116 (PMC5088568; doi:10.1101/gad.284992.116)
Supplement: Supplemental Material [file supp_30.19.2199_Supplementary_table5.pdf]

List of primers used in this study

RT-qPCR

Name	*Sequence*	*Source publication*
IAP-5'UTR-F	CGGGTCGCGGTAATAAAGGT	Rowe et al. 2010
IAP-5'UTR-R	ACTCTCGTTCCCCAGCTGAA	
LINE-1-ORF1-F	GAACCAAGACCACTCACCATCA	Vitullo et al. 2012
LINE-1-ORF1-R	CCCTGGACTGGGCGAAGT	
SINE-B1-F	TGGCGCACGCCTTTAATC	Gualtieri et al. 2013
SINE-B1-R	TGGCCTCGAACTCAGAATCC	
Uhrf1-F	AAACGCCCTGAGTTTTCGC	Designed in this study
Uhrf1-R	GCCGATGTACTCTCTCACG	
Gapdh-F	ATTCAACGGCACAGTCAAGG	Designed in this study
Gapdh-R	TGGATGCAGGGATGATGTTC	

Oxidative bisulfite sequencing

IAP GAG-F	AGGTTAGTTTGTTGATTGGTTTTAG	Sadic et al. 2015
IAP GAG-R	AATCAACAAAATAAACTCCCTAACC	
LINE-1-F	GTTAGAGGATTTGATAGTTTTTGGAATAGG	Tommasi et al. 2012
LINE-1-R	CCAAAACAAAACCTTTCTCAAACACTATAT	
