# Supplementary material for: Loss of Uhrf1 in neural stem cells leads to activation of retroviral elements and delayed neurodegeneration
Source: Genes Dev. 2016 Oct 1;30(19):2199–212. doi: 10.1101/gad.284992.116 (PMC5088568; doi:10.1101/gad.284992.116)
Supplement: Supplemental Material [file supp_30.19.2199_Supplementary_fig8.pdf]

Ramesh_Supplementary figure 8

A Overlap of hypomethylated and upregulated genes

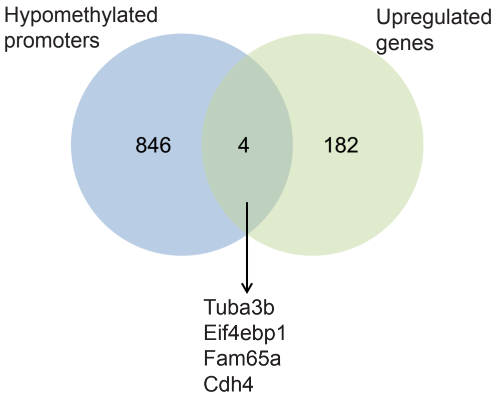

B LINE-1 - 5mC against mRNA changes - E16

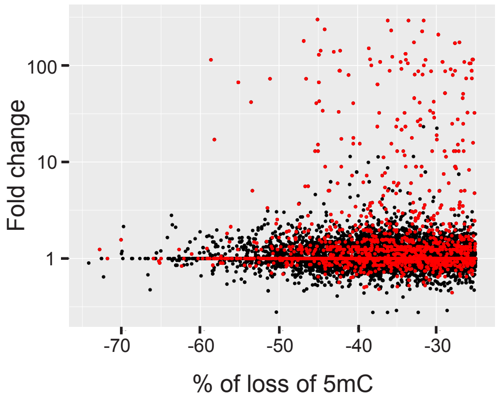

C Loss of 5mC at LINE-1 regulatory regions

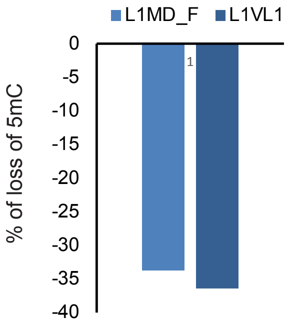

D Repeat classes with high 5hmC

IAPEY_LTR|LTR|ERVK
IAPA_MM-int|LTR|ERVK
IAPLTR1_Mm|LTR|ERVK
IAPLTR1a_Mm|LTR|ERVK
