# Supplementary material for: Loss of Uhrf1 in neural stem cells leads to activation of retroviral elements and delayed neurodegeneration
Source: Genes Dev. 2016 Oct 1;30(19):2199–212. doi: 10.1101/gad.284992.116 (PMC5088568; doi:10.1101/gad.284992.116)
Supplement: Supplemental Material [file supp_30.19.2199_Supplementary_table2.pdf]

Ramesh_Supplementary table 2

RNASeq - P5

Upregulated genes

Gene name	P-value	FC	Gene name	P-value	FC	Gene name	P-value	FC
Rhox5	0.00195	65.47	Apoc1	0.00035	3.09	Ogn	0.00205	2.29
Gm773	5.00E-05	20.63	Bfsp1	5.00E-05	3.07	Igtp	5.00E-05	2.29
Xlr3c	5.00E-05	15.91	Tdrd9	5.00E-05	3.00	Tcfl5	0.00235	2.28
AI662270	5.00E-05	14.89	Cd74	5.00E-05	2.99	Pgam2	5.00E-05	2.28
E330017A01Rik	5.00E-05	13.67	Wfdc12	5.00E-05	2.99	Prss45	0.00215	2.28
Slc47a1	5.00E-05	11.09	A330049N07Rik	0.00405	2.98	Olfr1054	5.00E-05	2.28
Uba1y	0.00015	9.34	Piwil2	5.00E-05	2.95	Gm11413	5.00E-05	2.27
Cox6a2	5.00E-05	9.33	Atp6ap1l	5.00E-05	2.93	Bst2	5.00E-05	2.26
Mnd1	5.00E-05	9.10	Magea10	5.00E-05	2.90	Pnpt1	5.00E-05	2.24
Pet2	5.00E-05	7.97	Atf5	0.00025	2.90	Psph	5.00E-05	2.24
Sectm1a	5.00E-05	7.71	Efcab6	5.00E-05	2.89	Cilp2	5.00E-05	2.21
Apoh	5.00E-05	7.59	Hsf2bp	5.00E-05	2.89	Ddr2	5.00E-05	2.21
4930500J02Rik	5.00E-05	7.31	Lgals9	5.00E-05	2.89	Arhgap30	5.00E-05	2.21
Myl4	5.00E-05	6.85	Gm6268	5.00E-05	2.88	Psmb8	5.00E-05	2.20
Hamp2	5.00E-05	6.71	Rabggta	5.00E-05	2.86	H2-Aa	0.00045	2.20
Ccnb1ip1	5.00E-05	6.58	Cyb5r1	5.00E-05	2.86	Gm13826	5.00E-05	2.19
Cox7b2	0.00325	6.51	Rhox2a	5.00E-05	2.85	Adad2	0.00385	2.18
Slc7a3	5.00E-05	6.27	Olfr194	5.00E-05	2.84	Hk2	5.00E-05	2.16
Gpat2	2.00E-04	6.04	Mia	5.00E-05	2.79	Foxs1	5.00E-05	2.16
Kntc1	5.00E-05	6.03	Col6a3	5.00E-05	2.78	Mbl2	5.00E-05	2.15
Il15	5.00E-05	5.99	Dpep1	5.00E-05	2.71	Eda2r	5.00E-05	2.15
Ptgds	5.00E-05	5.81	Tmem184a	5.00E-05	2.70	Dhrs3	5.00E-05	2.12
Ptprh	5.00E-05	5.61	H2-K1	5.00E-05	2.70	Pnma5	1.00E-04	2.11
Eif4ebp1	5.00E-05	5.24	Ly86	5.00E-05	2.68	AI848285	0.00015	2.10
Gpr97	5.00E-05	5.00	Arhgef18	5.00E-05	2.67	H19	5.00E-05	2.09
Tuba3b	5.00E-05	4.99	Plb1	5.00E-05	2.67	Isg15	0.0015	2.09
Cep112	5.00E-05	4.81	Sesn2	5.00E-05	2.67	Serpinb1a	5.00E-05	2.05
Trpc6	5.00E-05	4.61	H2-Ab1	5.00E-05	2.63	Eif2s3y	5.00E-05	2.04
Xlr3a	5.00E-05	4.60	Npy	5.00E-05	2.57	C1qb	5.00E-05	2.03
1700017N19Rik	5.00E-05	4.54	Capn11	5.00E-05	2.55	2410006H16Rik	5.00E-05	2.02
Mrgprx2	5.00E-05	4.51	Ifitm3	5.00E-05	2.53	Acpp	5.00E-05	2.01
Sema3b	5.00E-05	4.42	Nupr1	1.00E-04	2.52	Gpc3	5.00E-05	2.01
Chac1	5.00E-05	4.41	Tspo	5.00E-05	2.51	Rhox2d	5.00E-05	2.01
Tfpi	5.00E-05	4.34	Irgm1	5.00E-05	2.48	Hmx1	5.00E-05	2.01
Wfdc9	5.00E-05	4.34	Slc6a13	5.00E-05	2.47	Mmp21	5.00E-05	2.01
6720468P15Rik	5.00E-05	4.14	Ccdc3	5.00E-05	2.47			
Ugt3a2	5.00E-05	4.11	Pnldc1	5.00E-05	2.46			
Mnd1-ps	5.00E-05	4.10	Pnmt	0.00025	2.46			
Gbgt1	5.00E-05	4.02	Avil	5.00E-05	2.44			
Mageb4	5.00E-05	3.77	Mfap5	5.00E-05	2.42			
Suv39h2	5.00E-05	3.77	Svs5	5.00E-05	2.42			
Xlr3b	5.00E-05	3.77	Gpr98	5.00E-05	2.41			
Ccrn4l	5.00E-05	3.76	Igf2	5.00E-05	2.40			
Cdh19	5.00E-05	3.40	Mgp	1.00E-04	2.39			
E330020D12Rik	0.0035	3.24	Grhl2	1.00E-04	2.36			
Ifi27	5.00E-05	3.24	Ddit3	0.0034	2.36			
Dcn	5.00E-05	3.15	Cdkn1c	5.00E-05	2.35			
Pdlim2	5.00E-05	3.14	B2m	5.00E-05	2.32			
Stc2	5.00E-05	3.14	Olfr193	5.00E-05	2.32			
Mitf	5.00E-05	3.13	1700048O20Rik	5.00E-05	2.32			
Mthfd2	5.00E-05	3.13	Lyz2	5.00E-05	2.31			
Cdkn2b	5.00E-05	3.13	H2bfm	5.00E-05	2.29			

Downregulated genes

Gene name	P-value	FC
Tshz2	5.00E-05	0.14
Nefl	5.00E-05	0.16
Lefty2	5.00E-05	0.24
Fam163a	5.00E-05	0.28
Nefm	5.00E-05	0.28
Gpr88	5.00E-05	0.30
Scd1	5.00E-05	0.31
Rprml	5.00E-05	0.35
Nxnl2	0.00015	0.40
Nts	5.00E-05	0.40
Ntf3	5.00E-05	0.40
Gsto1	5.00E-05	0.42
Crym	5.00E-05	0.42
Sowaha	5.00E-05	0.42
Itpka	5.00E-05	0.42
Grp	5.00E-05	0.42
Pcbd1	5.00E-05	0.43
Gstt1	5.00E-05	0.45
Resp18	5.00E-05	0.45
Met	5.00E-05	0.45
Lynx1	5.00E-05	0.45
Slc16a11	5.00E-05	0.46
Rxrg	5.00E-05	0.46
Zdbf2	5.00E-05	0.46
Trim16	5.00E-05	0.47
Plcd3	5.00E-05	0.47
Rasgrf1	1.00E-04	0.47
Fam132a	5.00E-05	0.47
Tmem212	0.0019	0.47
Lmo4	5.00E-05	0.48
Cartpt	0.00015	0.48
Krt222	5.00E-05	0.49
Phyhip	5.00E-05	0.49
Mchr1	5.00E-05	0.49
Tekt5	5.00E-05	0.49
2010300C02Rik	5.00E-05	0.49
Coro2a	5.00E-05	0.50
Arhgef15	5.00E-05	0.50
Slc10a4	5.00E-05	0.50
Ntng2	1.00E-04	0.50
